# Regulation of Gramicidin Channel Function Solely by Changes in Lipid Intrinsic Curvature

**DOI:** 10.3389/fphys.2022.836789

**Published:** 2022-03-08

**Authors:** Andreia M. Maer, Radda Rusinova, Lyndon L. Providence, Helgi I. Ingólfsson, Shemille A. Collingwood, Jens A. Lundbæk, Olaf S. Andersen

**Affiliations:** Department of Physiology and Biophysics, Weill Cornell Medical College, New York, NY, United States

**Keywords:** electrostatic interactions, single-channel, fluorescence quench, intrinsic curvature, continuum elastic model, bilayer deformation energy, gramicidin channels, elasticity

## Abstract

Membrane protein function is regulated by the lipid bilayer composition. In many cases the changes in function correlate with changes in the lipid intrinsic curvature (*c*_0_), and *c*_0_ is considered a determinant of protein function. Yet, water-soluble amphiphiles that cause either negative or positive changes in curvature have similar effects on membrane protein function, showing that changes in lipid bilayer properties other than *c*_0_ are important—and may be dominant. To further investigate the mechanisms underlying the bilayer regulation of protein function, we examined how maneuvers that alter phospholipid head groups effective “size”—and thereby *c*_0_—alter gramicidin (gA) channel function. Using dioleoylphospholipids and planar bilayers, we varied the head groups’ physical volume and the electrostatic repulsion among head groups (and thus their effective size). When 1,2-dioleyol-sn-glycero-3-phosphocholine (DOPC), was replaced by 1,2-dioleyol-sn-glycero-3-phosphoethanolamine (DOPE) with a smaller head group (causing a more negative *c*_0_), the channel lifetime (τ) is decreased. When the pH of the solution bathing a 1,2-dioleyol-sn-glycero-3-phosphoserine (DOPS) bilayer is decreased from 7 to 3 (causing decreased head group repulsion and a more negative *c*_0_), τ is decreased. When some DOPS head groups are replaced by zwitterionic head groups, τ is similarly decreased. These effects do not depend on the sign of the change in surface charge. In DOPE:DOPC (3:1) bilayers, pH changes from 5→9 to 5→0 (both increasing head group electrostatic repulsion, thereby causing a less negative *c*_0_) both increase τ. Nor do the effects depend on the use of planar, hydrocarbon-containing bilayers, as similar changes were observed in hydrocarbon-free lipid vesicles. Altering the interactions among phospholipid head groups may alter also other bilayer properties such as thickness or elastic moduli. Such changes could be excluded using capacitance measurements and single channel measurements on gA channels of different lengths. We conclude that changes gA channel function caused by changes in head group effective size can be predicted from the expected changes in *c*_0_.

## Introduction

Membrane proteins are coupled to their host bilayer through hydrophobic interactions, and the need for hydrophobic adaptation, or matching, between lipid bilayers and the embedded membrane proteins (Israelachvili, 1977; Mouritsen and Bloom, 1984) causes protein conformational transitions involving the protein-bilayer interface to perturb the surrounding bilayer. Such bilayer perturbations incur energetic costs (Huang, 1986; Helfrich and Jakobsson, 1990; Ring, 1996; Nielsen et al., 1998; May, 2000; Nielsen and Andersen, 2000) that contribute to the free energy difference of the protein conformational changes (Gruner, 1991; Lundbæk et al., 2010; Rusinova et al., 2011). The bilayer contribution to the deformation energy varies as a function of the bilayer collective (or material) properties—bilayer hydrophobic thickness (*d*_0_), lipid intrinsic curvature (*c*_0_) and the bilayer elastic moduli (e.g., Nielsen and Andersen, 2000; Lundbæk et al., 2010), which are functions of bilayer composition. The hydrophobic coupling between membrane proteins and their host bilayer thus provides a mechanistic basis for the bilayer regulation of protein function (Sackmann, 1984; Andersen et al., 1992; Brown, 1994; Partenskii and Jordan, 2002; Andersen and Koeppe, 2007; Marsh, 2008; Rusinova et al., 2021).

**FIGURE 1:**
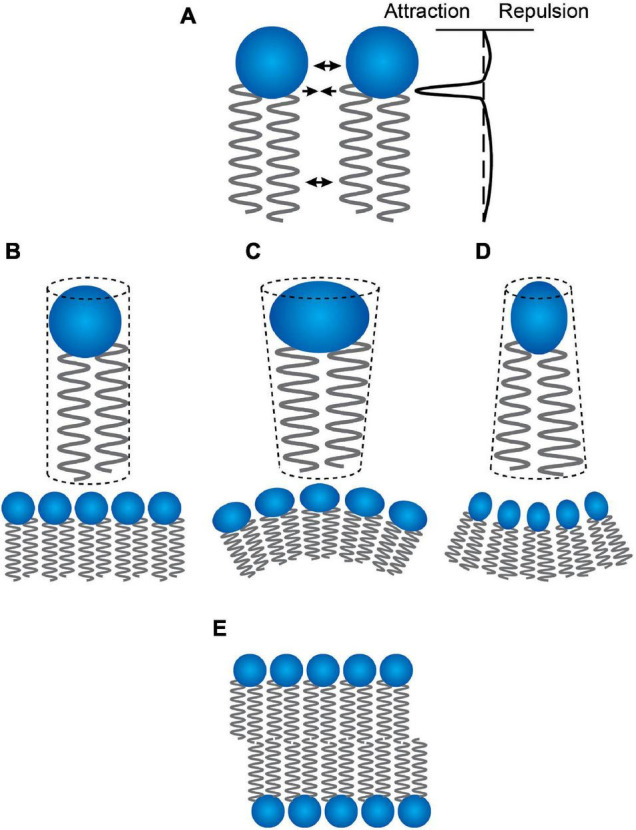
Lipid “shape” and curvature. **(A)** the profile of intermolecular interactions between two lipid molecules in a bilayer. Changes in this profile alter the effective lipid “shape” and the intrinsic curvature, *c*_0_, of the bilayer-forming lipids. **(B)** Lipids that have a cylindrical shape form plane monolayers with no intrinsic curvature. **(C)** Increased repulsion between the head groups will cause the lipids to be cone-shaped with the broad base toward the aqueous solution—and to form monolayers with a positive curvature (the surface is convex when viewed from the aqueous solution). **(D)** Decreased repulsion between the head groups will cause the lipids to be cone-shaped with the broad base toward the terminal methyl groups—and to form monolayers with a negative curvature (the surface is concave when viewed from the aqueous solution). **(E)** As long as the curvature is not too extreme, all three types of lipids can form planar bilayers—with the bilayers formed by lipids having intrinsic curvature being under a curvature frustration stress, which will contribute the energetics of channel formation (the energetic cost of a channel-imposed bilayer thinning will decrease as *c*_0_ increases and increase as *c*_0_ decreases).

The curvature of an isolated, relaxed monolayer (the lipid intrinsic curvature Gruner, 1985) reflects the profile of attractive and repulsive forces between the constituent lipid molecules (Helfrich, 1981; Seddon, 1990; Andersen and Koeppe, 2007; Marsh, 2007). Attractive forces (due to hydrophobic interactions) at the hydrocarbon-water interface tend to decrease the contact area between acyl chains and water; repulsive forces between the head groups (due to steric or electrostatic interactions or changes in hydration) or the acyl chains (due to thermal motion) tend to increase the interfacial area exposed to water (Figure 1A). In an isolated relaxed monolayer the intermolecular force profile determines the effective cross-sectional areas of head groups and acyl chains, which in turn define geometric packing constraints (Israelachvili et al., 1977) and a molecular “shape” (Cullis and de Kruijff, 1979, cf. Figures 1B–D).

Cylindrical lipids (Figure 1B) will form planar surfaces and are classified as having have zero intrinsic curvature (*c*_0_ = 0). Cone-shaped lipids with head group cross-sectional areas that are greater than that of the acyl chains (Figure 1C) will form convex surfaces with positive curvatures (as viewed from the aqueous phase) and are therefore classified as having positive intrinsic curvatures (*c*_0_ > 0). Cone-shaped lipids with head group cross-sectional areas that are less than that of the acyl chains (so-called inverted cones, Figure 1D) will form concave surfaces with negative curvature and are therefore classified as having negative intrinsic curvatures (*c*_0_ < 0). In a symmetrical planar bilayer, attractive interactions between the monolayers oppose the tendency of the monolayers to adopt non-planar configurations. Whenever *c*_0_ ≠ 0 the bilayer will be planar (Figure 1E) but under a state of curvature stress (Gruner, 1985).

Membrane protein function is regulated by experimental manipulations that alter *c*_0_ of the bilayer-forming lipids or, equivalently, alter the profile of intermolecular interactions across the bilayer (Helfrich, 1981; Seddon, 1990; Cantor, 1997; Andersen and Koeppe, 2007; Marsh, 2007), and *c*_0_ is considered a determinant of membrane protein function (Tate et al., 1991; Brown, 1994; Bezrukov, 2000; Epand et al., 2015). Yet, uncertainties remain. *c*_0_ varies with the phospholipid head group composition and can be changed by replacing choline with ethanolamine head groups (Gruner et al., 1988; Rand and Parsegian, 1997), but at least some of the changes in protein function may result from specific lipid-protein interactions (Hakizimana et al., 2008; Bondar et al., 2009; Martens et al., 2016). Amphiphiles may also alter *c*_0_ (e.g., Lundbæk et al., 2005), but amphiphiles that produce either negative or positive changes in *c*_0_ have similar effects on the function of gramicidin (gA) channels (Lundbæk et al., 2005; Bruno et al., 2007), voltage-dependent sodium channels (Leaf et al., 2002; Lundbæk et al., 2005), and GABA_*A*_ receptors (Søgaard et al., 2006; Chisari et al., 2010), see also (Lundbæk, 2006; Lundbæk et al., 2010). To further investigate the regulation of membrane protein function by the bilayer material properties, we examine how maneuvers that alter the effective size of phospholipid head groups affect gA channel function and compare the results to predictions based on changes in *c*_0_ or bilayer elastic moduli.

**FIGURE 2:**
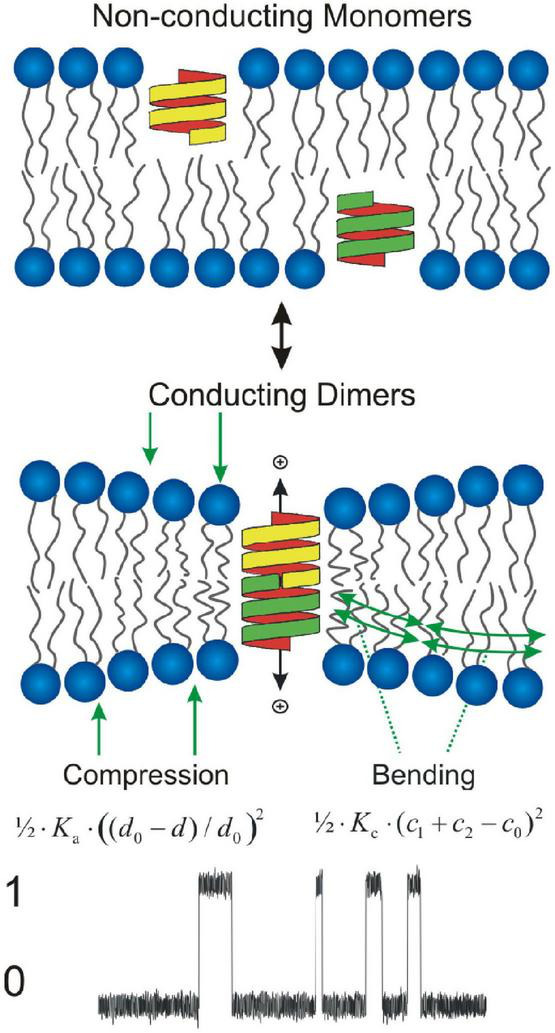
Gramicidin channels as molecular force probes. Gramicidin channels form by transmembrane association of b^6⋅3^-helical monomers imbedded in each monolayer, which is associated with compression and bending of the two bilayer leaflets toward each other. The local bilayer deformation imposed by the hydrophobic mismatch between the channel and its host bilayer, produces a compression of (each leaflet of) the bilayer with an energy density 1/2⋅*K*_a_⋅((*d*_0_−*d*)/*d*_0_)^2^, where *K*_*a*_ denotes the elastic area-compression modulus, *d*_0_ the thickness of the unperturbed bilayer, and *d* the local bilayer thickness as function of distance from the channel, and a bending of the each monolayer/solution interface with an energy density we approximate as 1/2⋅*K*_c_⋅(*c*_1_ + *c*_2_−*c*_0_)^2^ where *c*_1_ and *c*_2_ denote the principal components of the local curvature. Bottom: Single channel current transitions associated with channel formation/dissociation (0: no channel; 1: conducting channel).

gA channels (Andersen et al., 2007) are membrane mini-proteins formed by the transbilayer dimerization of subunits residing in the opposing monolayers (O’Connell et al., 1990; Figure 2).

gA channels have a near-cylindrical shape with a radius (*r*_0_ ≈ 1.0 nm); their hydrophobic length (*l* ≈ 2.2 nm, for channels formed by 15-residue gramicidins) is less than the average hydrophobic thickness of an unperturbed bilayer (*d*_0_ ≈ 4 nm for hydrocarbon-containing bilayers). The hydrophobic coupling between the channel and its host bilayer, cause channel formation to produce a bilayer perturbation (deformation), where the bilayer hydrophobic thickness locally adjusts to match the channel hydrophobic length (Figure 2). This hydrophobic adaptation incurs an energetic cost (Huang, 1986; Nielsen and Andersen, 2000), the bilayer deformation energy ($\Delta \,G_{\text{def}}^{\text{D}}$) that results from compression of the bilayer and bending of the bilayer/solution interface adjacent to the conducting dimeric channel (Figure 2). The free energy of channel formation (Δ*G*^M→D^) therefore will be given by


(1) $$\Delta \,G^{\text{M}\to \text{D}}=\Delta \,G_{\text{protein}}^{\text{M}\to \text{D}}+\Delta \,G_{\text{bilayer}}^{\text{M}\to \text{D}},$$

where $\Delta \,G_{\text{bilayer}}^{\text{M}\to \text{D}}\;\left(=\Delta \,G_{\text{def}}^{\text{D}}-2\cdot \Delta \,G_{\text{def}}^{\text{M}}\right)$ is the difference in bilayer deformation energy between the non-conducting monomers (M) and conducting dimers (D) and $\Delta \,G_{\text{protein}}^{\text{M}\to \text{D}}$ the energetic contributions from the dimerization *per se* (Lundbæk et al., 2010; Sun et al., 2020).

Lipid bilayers are elastic bodies (e.g., Helfrich, 1973; Evans and Hochmuth, 1978), and the local bilayer deformation caused by a membrane protein-induced bilayer deformation (Figure 2) has an energetic cost that can decomposed into: a bilayer compression with an energy density per unit area 1/2⋅*K*_a_⋅((*d*_0_−*d*)/*d*_0_)^2^, where *K*_*a*_ denotes the elastic area-compression modulus (units: energy/area) and *d* is the local bilayer thickness; and a bending of the bilayer/solution interfaces with an energy density that can be approximated as 1/2⋅*K*_c_⋅(*c*_1_ + *c*_2_−*c*_0_)^2^, where *K*_*c*_ denotes the elastic bending modulus (units: energy) and *c*_1_ and *c*_2_ are the principal components of the local curvature. For a cylindrical protein (membrane inclusion) with radius *r*_0_, the bilayer deformation energy can then can be obtained using a continuum elastic model (Huang, 1986; Helfrich and Jakobsson, 1990; Nielsen and Andersen, 2000) by integrating the energy densities over the perturbed area:


(2) $$\Delta \,G_{\text{def}}^{\text{D}}=\frac{1}{2}\cdot \int_{r_{0}}^{\infty }\left(K_{\text{a}}\cdot {\left(\frac{d_{0}-d}{d_{0}}\right)}^{2}+K_{\text{c}}\cdot {\left(c_{1}+c_{2}-c_{0}\right)}^{2}\right)\cdot 2\,\pi \cdot r\,\text{d}\,r-\frac{1}{2}\cdot \int_{r_{0}}^{\infty }K_{\text{c}}\cdot c_{0}^{2}\cdot 2\,\pi \cdot r\,\text{d}\,r$$

$$=K_{\text{a}}\cdot \int_{r_{0}}^{\infty }{\left(\frac{d_{0}-d}{d_{0}}\right)}^{2}\cdot \pi \cdot r\,\text{d}\,r+K_{\text{c}}\cdot \int_{r_{0}}^{\infty }\left({\left(c_{1}+c_{2}\right)}^{2}-\left(c_{1}+c_{2}\right)\cdot c_{0}\right)\cdot \pi \cdot r\,\text{d}\,r,$$

where $K_{\text{c}}\cdot c_{0}^{2}/2$ is the curvature frustration energy density in the unperturbed bilayer and solving for the bilayer thickness profile that minimizes the energy (see Nielsen et al., 1998; Nielsen and Andersen, 2000) for details.

Despite its seeming complexity Eq. 2 can be expressed analytically in terms of the bilayer-channel hydrophobic mismatch, *d*_0_ – *l*, and *c*_0_ (Nielsen and Andersen, 2000):


(3) $$\Delta \,G_{\text{def}}^{\text{D}}\,\left(d_{0}-l,c_{0}\right)=H_{\text{B}}\cdot {\left(d_{0}-l\right)}^{2}+H_{\text{X}}\cdot \left(d_{0}-l\right)\cdot c_{0}+H_{\text{C}}\cdot c_{0}^{2},$$

where the three elastic coefficients (*H*_*B*_, *H*_*X*_, and *H*_*C*_) are functions of *K*_*a*_ and *K*_*c*_, *d*_0_, and *r*_0_, but not *c*_0_, which is included through the boundary conditions for matching the bilayer to the channel at *r*_0_ (Nielsen and Andersen, 2000). (Even when *c*_0_ = 0, and Eq. 3 reduces to that for a simple linear spring, $\Delta \,G_{\text{def}}^{\text{D}}\,\left(d_{0}-l,0\right)=H_{\text{B}}\cdot {\left(d_{0}-l\right)}^{2}$, the phenomenological spring coefficient *H*_*B*_ is a function of both *K*_*a*_ and *K*_*c*_ because the bilayer adaptation to the channel involves both compression and bending, Figure 2.) $\Delta \,G_{\text{def}}^{\text{M}}$ is often considered to be 0, but the monomers will perturb the adjacent lipids in each leaflet (Beaven et al., 2017b; Sodt et al., 2017), and $\Delta \,G_{\text{def}}^{\text{M}}$ is likely to be non-zero.

Because the deformation energy has contributions from both the compression-expansion of the bilayer and the bending of the bilayer/solution interface, $\Delta \,G_{\text{def}}^{\text{D}}$ can also be approximated as a Taylor expansion in (*d*_0_ – *l*) and *c*_0_ (Andersen and Koeppe, 2007; Rusinova et al., 2011):


$$\Delta \,G_{\text{def}}^{\text{D}}\,\left(d_{0}-l,c_{0}\right)=\Delta \,G_{\text{def}}^{\text{D}}\,\left(0,0\right)+\frac{\partial \left(\Delta \,G_{\text{def}}^{\text{D}}\right)}{\partial \left(d_{0}-l\right)}\cdot \left(d_{0}-l\right)++\frac{\partial \left(\Delta \,G_{\text{def}}^{\text{D}}\right)}{\partial c_{0}}\cdot c_{0}+\frac{1}{2}\,\frac{\partial^{2}\left(\Delta \,G_{\text{def}}^{\text{D}}\right)}{\partial {\left(d_{0}-l\right)}^{2}}\cdot {\left(d_{0}-l\right)}^{2}$$

(4) $$+\frac{\partial^{2}\left(\Delta \,G_{\text{def}}^{\text{D}}\right)}{\partial \left(d_{0}-l\right)\,\partial c_{0}}\cdot \left(d_{0}-l\right)\cdot c_{0}+\frac{1}{2}\cdot \frac{\partial^{2}\left(\Delta \,G_{\text{def}}^{\text{D}}\right)}{\partial c_{0}^{2}}\cdot c_{0}^{2}+\ldots ,$$

where the first-order derivatives will be zero because the deformation energy for small decreases in (*d*_0_ − *l*) should equal that for small increases of equal magnitude, which requires $\partial \left(\Delta \,G_{\text{def}}^{\text{D}}\right)/\partial \left(d_{0}-l\right)$ to be zero, with a similar reasoning holding for $\partial \left(\Delta \,G_{\text{def}}^{\text{D}}\right)/\partial c_{0}$. Eq. 4 should be valid for arbitrary membrane protein geometries, and $\Delta \,G_{\text{def}}^{\text{D}}\,\left(0,0\right)$ incorporates the loss of conformational entropy of the acyl chains adjacent to the channel (Fattal and Ben-Shaul, 1993), which is not considered in the continuum elastic model [$\Delta \,G_{\text{def}}^{\text{D}}\,\left(0,0\right)=0$ in Eq. 3]. Comparing Eqs 3 and 4, we can equate the *H* coefficients with the second-order


(5) $$H_{\text{B}}=\frac{1}{2}\cdot \frac{\partial^{2}\left(\Delta \,G_{\text{def}}^{0}\right)}{\partial {\left(l-d_{0}\right)}^{2}},H_{\text{X}}=\frac{\partial^{2}\left(\Delta \,G_{\text{def}}^{0}\right)}{\partial \left(l-d_{0}\right)\,\partial c_{0}},\text{and}\,H_{\text{C}}=-\frac{1}{2}\cdot \frac{\partial^{2}\left(\Delta \,G_{\text{def}}^{0}\right)}{\partial c_{0}^{2}},$$

that is the simplicity of Eq. 3, which becomes important for the application to experimental results, should apply also to more complex membrane protein shapes.

The bilayer responds to a gramicidin channel-induced deformation by imposing a disjoining force (*F*_dis_) on the channel that will tend to pull the dimer apart (Rusinova et al., 2011):


(6) $$F_{\text{dis}}=-\frac{d\,\left(\Delta \,G_{\text{def}}^{\text{D}}\right)}{d\,\left(d_{0}-l\right)}=-2\cdot H_{\text{B}}\cdot \left(d_{0}-l\right)-H_{\text{X}}\cdot c_{0}.$$

The hydrophobic coupling between the channel and the host bilayer thus causes the monomer↔dimer equilibrium constant (*K*_*D*_), the channel appearance rate (*f*), and the lifetime (τ) to vary with changes in the channel bilayer hydrophobic mismatch (*d*_0_ –*l*) and intrinsic curvature *c*_0_. When *c*_0_ > 0, denoting a preference of a leaflet to form a convex surface (Figure 1C), the local bilayer thinning associated with channel formation will be promoted and the monomer↔dimer will shift toward the right; when *c*_0_ < 0, denoting a preference of a leaflet to form a concave surface (Figure 1D), the local bilayer thinning associated with channel formation will be impeded and the monomer↔dimer will shift toward the left.

To examine how changes in the lipid head group interactions (which will change *c*_0_) alter gA channel function, we varied the head group steric volume and electrostatic interactions, which are known to alter *c*_0_ (Anderson et al., 1988; Rand and Parsegian, 1997; May, 2000) and thereby the curvature frustration energy density $K_{\text{c}}\cdot c_{0}^{2}/2$ in the bilayer. Changes in head group interactions alter gA channel function in a manner that would be expected from isolated changes in *c*_0_. Comparing the changes in lifetimes for channels of different lengths allows for separating the *H*_B_⋅(*d*_0_−*l*) and *H*_X_⋅*c*_0_ terms in Eq. 6, because a lack of difference indicates no change in *H*_*B*_. That is, the bilayer elastic moduli were not altered by changes in head group interactions, indicating that the primary driver of the changes in channel function was the changes in curvature. These results provide additional insight into the regulation of membrane protein function by hydrophobic coupling to the host lipid bilayer. They also demonstrate the power of using gA channels to probe the energetic contributions to the bilayer deformation energy associated with a protein conformational change.

## Materials and Methods

### Electrophysiological Experiments

#### Materials

1,2-dioleyol-sn-glycero-3-phosphocholine (DOPC), 1,2-dioleyol-sn-glycero-3-phosphoethanolamine (DOPE), 1,2-dioleyol-sn-glycero-3-phosphoserine (DOPS), the ether phospholipid 1,2-di-O-(9Z-octadecenyl)-sn-glycero-3-phosphocholine (DoPC) and the di-unsaturated 1,2-dilinoleoyl-sn-glycero-3-phosphocholine (DLoPC) were from Avanti Polar Lipids Inc. (Alabaster, AL). HPLC-purified [Val^1^]gA [VgA(15)] and the gA analogs [Ala^1^]gA [AgA(15)] and des-(Val^1^-Gly^2^)gA^–^ [gA^–^(13)] were gifts from Dr. R.E. Koeppe II, Univ. of Arkansas. *n*-decane (99.9% pure) was from ChemSampCo (Trenton, NJ). NaCl was Suprapur grade from EMD Industries (Gibbstown, NJ). HEPES, Na_2_HPO_4_, glycyl-glycine (Gly-Gly), and EDTA were from Sigma Chemical Co. (St. Louis, MO).

VgA(15), AgA(15), and gA^–^(13) were prepared as 1–10 nM stock solutions in ethanol and added to the electrolyte solution to nominal concentrations of 0.1–10 pM. Bilayer-forming solutions of DOPC, DOPE, DOPS, DoPC, and DLoPC were prepared in *n*-decane at 2–3% wt/vol. (For the experiments with DLoPC, the bilayer-forming solution was stabilized with 25 μM butylated hydroxytoluene.) The intrinsic pK values of the different head groups are listed in Table 1A.

**TABLE 1A:** pK values for the phospholipid head groups used in this study.

Phospholipid	pK_1_ (Phosphate)	pK_2_ (Carboxylate)	pK_3_ (Amino group)
DOPC	∼1		
DOPE	∼1		∼10
DOPS	∼1	∼3.5	∼10

*Based on Cevc et al. (1981), Tsui et al. (1986), and Tocanne and Teissie (1990).*

The solutions used in the electrophysiological experiments were prepared daily and titrated to the desired pH using 1.0 M solutions of NaOH or HCl (Mallinckrodt Baker Inc., Paris, KY). The compositions were as listed in Table 1B. The single-channel conductance and lifetime variations do not depend on the choice of buffers (HEPES, Na_2_HPO_4_, or Gly-Gly), which are “Good” buffers (Good et al., 1966; Pielak, 2021). At pH 7.0, the single-channel conductances and lifetimes were similar whether the buffer was HEPES or Na_2_HPO_4_. At pH 3.0 the conductances and lifetimes were similar whether the buffer was Gly-Gly or Na_2_HPO_4_.

**TABLE 1B:** Electrolyte solutions used in this study.

Experimental conditions	Salt	Buffer
DOPE:DOPC (0:1, 1:3, 1:1, 3:1)	1.0 M NaCl	Unbuffered
DOPS (pH 3–5)	0.1 M NaCl	10 mM Gly-Gly, 1 mM EDTA
DOPS (pH 6–8)	0.1 M NaCl	10 mM Na_2_HPO_4_, 1 mM EDTA
DOPS:DOPC (pH 7)	0.1 M NaCl	5 mM HEPES, 1 mM EDTA
DOPS:DOPE (pH 7)	0.1 M NaCl	5 mM HEPES, 1 mM EDTA
DOPS + divalent cations (pH 7)	0.1 M NaCl	5 mM HEPES
DOPE:DOPC (4:1) (pH 0–5)	0.1 M NaCl	10 mM Gly-Gly, 1 mM EDTA
DOPE:DOPC (4:1) (pH 7–10)	0.1 M NaCl	10 mM Na_2_HPO_4_, 1 mM EDTA
DoPC (pH 7)	1.0 M NaCl	10 mM HEPES
DLoPC (pH 7)	1.0 M NaCl	10 mM HEPES

#### Single-Channel Experiments

Single-channel experiments were done using the bilayer punch method (Andersen, 1983) at 25 ± 1°C, at an applied potential of ± 200 mV. The bilayers’ visible appearance did not vary as we varied the electrolyte solution: in all experiments the black lipid membrane was approximately circular with well-defined demarcation between bilayer and torus. The current signal was recorded and amplified using an AxoPatch 1B (Molecular Devices, Sunnyvale, CA) or a Dagan 3900A (Minneapolis, MN) patch clamp, filtered at 100–500 Hz, digitized and sampled by a PC/AT compatible computer. Single-channel current transitions were detected using an algorithm that detects current transitions (rather than current level crossings) as described previously (Andersen, 1983; Ingólfsson et al., 2008; Kapoor et al., 2008). Single-channel lifetimes were determined as described in Sawyer et al. (1989) and Ingólfsson et al. (2008), using a random number generator to match channel appearances to disappearances in case there were two, or more, simultaneously conducting channels. The average lifetimes were determined by fitting the results with single exponential distributions,


(7) N⁢(t)=N⁢(0)⋅exp⁢{-t/τ}

where τ is the average channel lifetime, *N*(0) the total number of channels and *N*(*t*) the number of channels with durations longer than *t*. Fitting was done using the non-linear least-squares routine in Origin (OriginLab, Northhampton, MA).

The effects of experimental maneuvers on the hydrophobic thickness of a lipid bilayer were monitored by measuring the specific bilayer capacitance (*C*_*m*_):


(8) $$d_{0}=\frac{\varepsilon_{0}\cdot \varepsilon_{\text{r}}}{C_{\text{m}}}$$

where ε_0_ and ε_*r*_ denote the permittivity of vacuum and the dielectric constant, respectively, using a sawtooth potential (±10 mV, 2.5 Hz) across the bilayer (Lundbæk and Andersen, 1994).

### Stopped-Flow Experiments

#### Materials

1,2-dierucoyl-sn-glycero-3-phosphoserine (DEPS) was from Avanti Polar Lipids (Alabaster, AL). The di-sodium salt of 8-aminonaphthalene-1,3,6-trisulfonate (ANTS) was from Invitrogen Life Technologies (Grand Island, NY). Thallium nitrate (TINO_3_) ≥ 99.9%, Sodium nitrate (NaNO_3_) ≥ 99%, HEPES ≥ 99.5%, and gramicidin from *Bacillus aneurinolyticus* (*Bacillus brevis*) ≥ 95% were from Sigma–Aldrich Co (St. Louis, MO).

#### Fluorescence Quench Experiments

The experiments were based on the use of thallium (Tl^+^) as a quencher of ANTS fluorescence (Ingólfsson and Andersen, 2010). Large unilamellar vesicles (LUVs) were made from DEPS following the procedures described in Ingolfsson et al. (2010) and Ingólfsson and Andersen (2010) for 1,2-dierucoyl-sn-glycero-3-phosphocholine. Briefly DEPS in chloroform was deposited into a round bottom flask, chloroform was dried off under Nitrogen gas flow. After desiccation overnight to remove the remaining chloroform, the lipid was rehydrated in 100 mM NaNO_3_, 25 mM ANTS, 10 mM HEPES, pH 7 for 4 h. The lipid suspension was sonicated and subjected to 6 freeze/thaw cycles, extruded with a mini extruder (Avanti Polar Lipids) through a 0.1 μm membrane, and the extravesicular ANTS was removed using a PD-10 desalting column (GE Healthcare, Piscataway, NJ) and stored in 140 mM, NaOH, 10 mM HEPES, pH 7. The LUVs then were incubated with gramicidin D for 24 h at 12°C before testing for channel activity by mixing the ANTS-loaded LUVs with a quench solution (50 mM TINO_3_ 94 mM NaNO_3_, 10 mM HEPES, pH 7) in a SX20 stopped-flow spectrofluorometer (Applied Photophysics, Leatherhead, United Kingdom). A fraction of LUVs prepared by extrusion using zwitterionic phosphatidylcholine are multilamellar (Scott et al., 2019). This problem is minimized by addition of negatively charged phospholipids (Scott et al., 2019), and we do not consider it further. The LUV size distribution and homogeneity was determined using dynamic light scattering using a Litesizer™ 500 and the Kalliope software (Anton Paar, Graz, Austria). The mean hydrodynamic diameter (*d*_*LUV*_), and the polydispersity index (PDI), defined as (σ/*d*_LUV_)^2^ (e.g., Clayton et al., 2016), where σ^2^ denotes the variance of the size distribution, were 120 nm and 0.01, respectively. The average radii of curvature of the inner and outer LUV leaflets, about ∓60 nm, were much larger in magnitude than the intrinsic radius of curvature of DOPS, ∼14 nm (Fuller et al., 2003), and were not considered further. (Fluctuations in vesicle shape e.g., Meleard et al., 1997, will cause both leaflets to undergo negative and positive changes in curvature.)

For a truly homogenous population of LUVs (same volume and gramicidin channel density in the membrane of each vesicle), the fluorescence quench traces will be described by simple exponential decays. Because of the unavoidable heterogeneity in LUV sizes, the rates of fluorescence quench were obtained by fitting the quench time course from each mixing reaction with a stretched exponential (Berberan-Santos et al., 2005; Ingólfsson and Andersen, 2010):


(9) $$F\,\left(t\right)=F\,\left(\infty \right)+\left(F\,\left(0\right)-F\,\left(\infty \right)\right)\cdot \exp\left\{-{\left(t/\tau_{0}\right)}^{\beta }\right\}$$

and evaluating the quench rate at 2 ms (the instrumental dead time is ∼1.5 ms):


(10) $$k\,\left(t\right)={\left(\beta /\tau_{0}\right)\cdot {\left(t/\tau_{0}\right)}^{\left(\beta -1\right)}|}_{2\,\text{ms}}$$

LUVs for 10 min at 25°C before acquiring quench time courses. Each measurement consisted of (4–8) individual mixing reactions, and the rates for each mixing reaction were averaged.

### Online Supplementary Material

The detailed derivation of the electrostatic energy of a charged monolayer in the presence of mono- and divalent ions that can bind to the head groups is available in Supplementary Material.

## Results

The relation between gA channel function and lipid intrinsic curvature was investigated by varying (a) the physical size of the lipid head groups; (b) the electrostatic repulsion among the head groups (and thus the effective head group size); and (c) by combining these maneuvers.

### Changing the Phospholipid Head Group Size

The relation between phospholipid head group volume and gA channel lifetime (τ) was studied in bilayers formed using different ratios of DOPC and DOPE (keeping the acyl chains the same). The van der Waals volumes of choline and ethanolamine are 0.101 and 0.063 nm^3^, respectively (Lundbæk and Andersen, 1994). Furthermore phosphatidylcholine head groups are more hydrated than phosphatidylethanolamine head groups (Elworthy, 1962; Rand and Parsegian, 1989). The intrinsic curvature of DOPE therefore is more negative than that of DOPC, which has almost zero curvature (Keller et al., 1993; Rand and Parsegian, 1997). Increasing the DOPE:DOPC ratio therefore will decrease the average head group size and cause a negative change in *c*_0_ (Gruner et al., 1988; Keller et al., 1993). The experiments were done using unbuffered solutions, pH 6–7, where the zwitterionic DOPE and DOPC head groups are neutral, such that only non-electrostatic head group interactions were altered.

**FIGURE 3:**
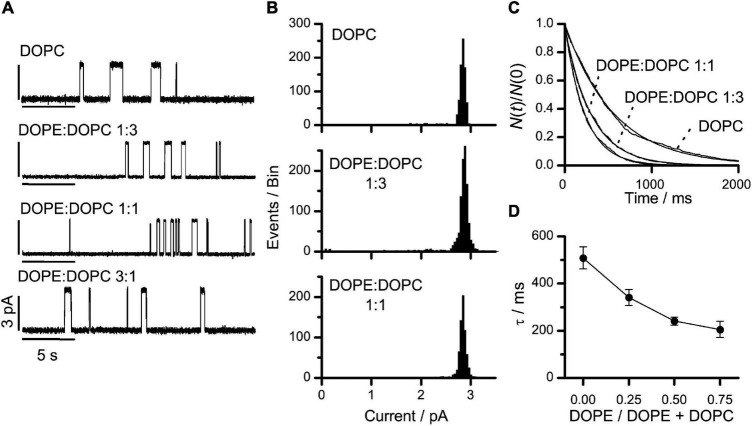
VgA(15) channel function in DOPE:DOPC/*n*-decane bilayers. **(A)** Current traces of VgA(15) channels in DOPE:DOPC/*n*-decane bilayers at different DOPE:DOPC molar ratios in the bilayer-forming solution. **(B)** Current transition amplitude histograms in DOPC and DOPE:DOPC (1:3 or 1:1) bilayers (results from single experiments). **(C)** Normalized single-channel duration histograms in DOPC (τ = 563 ms), DOPE:DOPC (1:3) (τ = 304 ms) or DOPE:DOPC (1:1) (τ = 229 ms) bilayers. Results from single experiments fitted by single exponential distributions. **(D)** Relation between DOPE/(DOPE + DOPC) and τ (mean ± SD, *n* ≥ 3). 1.0 M NaCl, 200 mV, 100 Hz.

Figure 3A shows single-channel current traces from experiments in DOPE:DOPC bilayers at DOPE:DOPC molar ratios from 0 (pure DOPC) to 3:1. Figures 3B,C show the corresponding current transition amplitude and lifetime histograms. At each experimental condition, only a single homogenous channel population was observed (Figures 3A–C). Increasing the DOPE:DOPC ratio from 0 to 3:1, caused about a 2.5-fold decrease in τ (Figure 3D). At DOPE:DOPC ratios above 3:1, the bilayers became increasing unstable, most likely because the DOPE-induced negative lipid intrinsic curvature leads to the formation of non-bilayer H_*II*_ phases (Cullis and de Kruijff, 1979; Kirk and Gruner, 1985).

The lifetime changes can be interpreted in terms of the kinetics of gA channel formation (Figure 2):


(Scheme 1) $$M+M\,\overset{k_{1}}{\underset{k_{-1}}{⇌}}\text{D,}$$

where *k*_1_ and *k*_–1_ (= 1/τ) are the association and dissociation rate constants, respectively (Bamberg and Läuger, 1973; Zingsheim and Neher, 1974; Veatch et al., 1975; Rokitskaya et al., 1996). Channel dissociation involves separating the channel-forming subunits by a distance δ≈ 0.16 nm to reach the transition state for dissociation (Durkin et al., 1993; Lundbæk and Andersen, 1999; Miloshevsky and Jordan, 2006; Greisen et al., 2011; Sun et al., 2021). Because the channel length in the transition state (*l*^‡^) larger than in the conducting state (*l*), the bilayer deformation (and the bilayer deformation energy) will be less for the transition state than for the conducting channel, and the difference in bilayer deformation energy ($\Delta \,G_{\text{bilayer}}^{‡,D\to \text{M}}=\Delta \,G_{\text{def}}^{‡,D\to \text{M}}-\Delta \,G_{\text{def}}^{\text{D}}$) will contribute to the activation energy for channel dissociation (Δ*G*^‡,*D*→*M*^):


(11) $$\Delta \,G^{‡,D\to \text{M}}=\Delta \,G_{\text{protein}}^{‡,D\to \text{M}}+\Delta \,G_{\text{bilayer}}^{‡,D\to \text{M}},$$

where $\Delta \,G_{\text{protein}}^{‡,D\to \text{M}}$ denotes the energetic contributions from the dimerization *per se*. The single-channel lifetime (τ) therefore can be expressed as Lundbæk and Andersen (1999):


(12) $$\ln\left\{\tau \right\}=-\ln\left\{k_{-1}\right\}=\ln\left\{\tau_{0}\right\}+\frac{\Delta \,G_{\text{protein}}^{‡,D\to \text{M}}+\Delta \,G_{\text{bilayer}}^{‡,D\to \text{M}}}{k_{\text{B}}\,T},$$

where 1/τ_0_ is a frequency factor for the reaction, *k*_*B*_ Boltzmann’s constant and *T* the temperature in Kelvin. When δ = *l*^‡^−*l*≪(*d*_0_−*l*), $\Delta \,G_{\text{bilayer}}^{‡,D\to \text{M}}$ can be approximated as (cf. Eq. 6):


(13) $$\Delta \,G_{\text{bilayer}}^{‡,D\to \text{M}}\approx \delta \cdot F_{\text{dis}}=-\delta \cdot \left(2\cdot H_{\text{B}}\cdot \left(d_{0}-l\right)+H_{\text{X}}\cdot c_{0}\right).$$

*H*_*X*_ is negative (as can be deduced from Nielsen and Andersen, 2000, Eq. 17), meaning that a negative change in *c*_0_ would be expected to increase the magnitude of *F*_*dis*_ (*F*_*dis*_ is directed away from the channel center and therefore negative) and thereby decrease $\Delta \,G_{\text{bilayer}}^{‡,D\to \text{M}}$, which in turn will decrease Δ*G*^‡,D→M^ and τ. A positive change in *c*_0_ would have the opposite effect. The lifetime changes we observe conform to these expectations.

### Changing the Electrostatic Interactions Among Phospholipid Head Groups

The intrinsic curvature, and phase-preference, of charged lipids can be varied by changing the electrostatic repulsion among the head groups, which changes the effective head group “size” (Tate et al., 1991; Lundbæk et al., 1997; Rand and Parsegian, 1997; May, 2000; Fuller et al., 2003). Previously, we showed that gA channel appearance rates and lifetimes in DOPS are decreased by addition of Ca^2+^ at concentrations ranging between 10^–6^ and 10^–3^ M (Lundbæk et al., 1997). Ca^2+^ reduces the electrostatic repulsion among the DOPS head groups by a combination of binding to the head groups and screening the surface charge (McLaughlin et al., 1981), which causes a negative *c*_0_. The effects of Ca^2+^ on DOPS are complex, however; though Ca^2+^ promotes negative changes in curvature (Cullis and Verkleij, 1979), increasing [Ca^2+^] does not promote a clear-cut lamellar→H_*II*_ phase transition (Rand, personal communication, quoted in Lundbæk et al., 1997).

To further investigate the relation between lipid head group electrostatic interactions and gA channel lifetime, we examined the effects of varying pH on gA channels in DOPS bilayers. Serine has a van der Waals volume of 0.083 nm^3^ (Lundbæk and Andersen, 1994), the phosphate, carboxyl and amino groups in DOPS have intrinsic pKs of ∼1, ∼3.5, and ∼10, respectively (Table 1A). At pH 7, the phosphate and carboxyl groups are fully deprotonated and negatively charged, and the amino group is fully protonated and positively charged—and the head groups’ net charge is −1. As pH is decreased the phosphate and carboxyl group become protonated. This reduces the electrostatic repulsion among the head groups (Papahadjopoulos, 1968) and thus the effective head group size, which, in turn, causes a negative change in *c*_0_ (Fuller et al., 2003).

**FIGURE 4:**
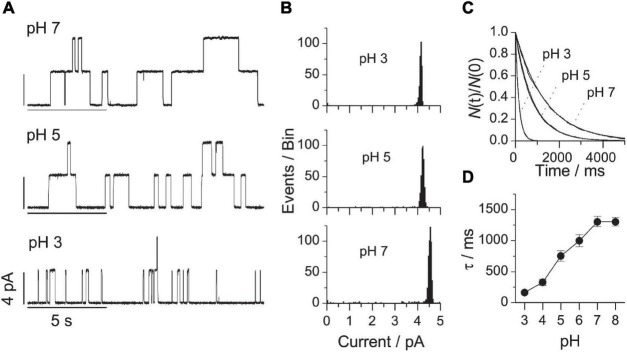
VgA(15) channel function in DOPS/*n*-decane bilayers at different pH. **(A)** Current traces of VgA(15) channels in DOPS/*n*-decane bilayers at pH: 7; 5 or 3. **(B)** Current transition amplitude histograms at pH 7, 5, or 3. **(C)** The corresponding normalized single-channel duration histograms. Results from single experiments fitted by single exponential distributions. **(D)** Relation between pH and channel lifetime (mean ± SD, *n* ≥ 3). 0.1 M NaCl buffered with either Gly-Gly or Na_2_HPO_4_, 200 mV, 100 Hz.

Figure 4A shows single-channel current traces obtained in DOPS bilayers at pH’s varying between 7 and 3. Figures 4B,C show the corresponding current transition amplitude and lifetime distributions. At each experimental condition, only a single channel population was observed (Figures 4A–C). Reducing pH from 7 to 3 caused a 10-fold decrease in τ (Figure 4D). There was no difference between the lifetimes at pH 7 and 8 (consistent with the observation that the surface potential of phosphatidylserine monolayers is invariant between pH 7 and 9; Papahadjopoulos, 1968). It was impossible to form stable bilayers at pH 2, most likely because of the tendency of DOPS to undergo a lamellar→H_*II*_ phase transition at low pH (Tate et al., 1991; Rostovtseva et al., 1998; Fuller et al., 2003).

Despite large changes in τ, the single-channel conductance varied little between pH 7 and 3, and there was no change in conductance between pH 5 and 3, where the lifetime varied more than fivefold across the same pH range (Figure 4B). The changes in τ and conductance therefore are not related. The conductance invariance is fortuitous, however. As shown by Rostovtseva et al. (1998), with Cs^+^ as the monovalent cation, when pH is reduced the protonation of DOPS leads to a less negative surface potential, which will decrease the interfacial alkali metal cation concentration and (its contribution to) the single-channel conductance. Due to gA channels’ high H^+^ permeability (Hladky and Haydon, 1972; Myers and Haydon, 1972; Rostovtseva et al., 1998), this happens to compensate for the reduced contribution from the alkali metal cation (*in casu*, Na^+^).

Again, the changes in pH (the DOPS head group charge) alter τ in a manner that conforms to expectations based on altered head group interactions and the ensuing changes in *c*_0_ (Eqs 12 and 13).

### Changing Both the Size of Head Groups and Their Electrostatic Interactions

We next investigated the effects of changing both the size of the lipid head groups and their electrostatic interactions. This was done using bilayers formed by DOPS:DOPE (1:2) or DOPS:DOPC (1:2) at pH 7, where DOPS is negatively charged but DOPE and DOPC are uncharged. In bilayers formed from either lipid mixture, the electrostatic repulsion among the head groups will be less than in pure DOPS bilayers, but the average, effective size of the head groups will be smaller in DOPS:DOPE than in DOPS:DOPC bilayers.

**FIGURE 5:**
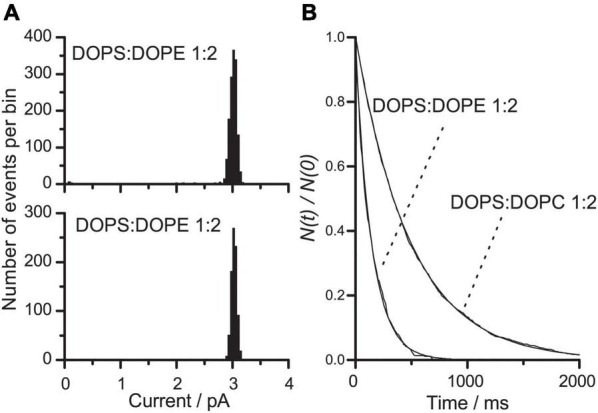
VgA(15) channel function in DOPS:DOPE/*n*-decane and DOPS:DOPC/*n*-decane bilayers. **(A)** Current transition amplitude histograms in DOPS:DOPE (1:2) or DOPS:DOPC (1:2) bilayers (results from single experiments). **(B)** The corresponding normalized single-channel duration histograms; in DOPS:DOPE (1:2) bilayers, τ = 143 ms; in DOPS:DOPC bilayers, τ = 486 ms. Results from single experiments fitted by single exponential distributions. 0.1 M NaCl pH 7 buffered with HEPES.

Figure 5 shows current transition amplitude and lifetime distributions for gA channels in DOPS:DOPE (1:2) or DOPS:DOPC (1:2). At each experimental condition, only a single channel population was observed. In both types of bilayers, the single-channel conductance was reduced by 30% relative to DOPS bilayers, indicating that the interfacial [Na^+^] and thus the surface charge densities are similar and less than in DOPS bilayers (compare Figures 4A, 5A).

The lifetimes of gA channels in DOPS:DOPE (1:2) or DOPS:DOPC (1:2) bilayers were decreased nine or threefold, respectively, compared to DOPS bilayers (Figure 5B). Again, the lifetime changes are not related to the changes in single-channel conductance. The larger effect of the DOPS→DOPE substitution, as compared to the DOPS→DOPC substitution, is expected because the smaller volume of the DOPE head group (as compared to DOPC head groups) would cause the lipid intrinsic curvature to be more negative.

### Changing Electrostatic Interactions Among Phosphatidylethanolamine Head Groups

To further probe the importance of the electrostatic interactions among the head groups, we exploited that DOPE has two titratable groups (Papahadjopoulos, 1968; Tocanne and Teissie, 1990, see also Table 1A). Therefore, as the pH is increased toward 10, the amino group deprotonates and the bilayer/solution interface becomes negatively charged; as pH is decreased toward 1, the phosphate group becomes protonated, such that the bilayer/solution interface becomes positively charged. The electrostatic repulsion among the head groups that occurs when pH is changed away from 4 to 6 toward either pH extreme will increase the effective area of the DOPE head groups, meaning that *c*_0_ becomes more positive as the pH is changed toward either extreme. The net surface charge density and interfacial potential, however, will increase as a monotonic function of pH.

**FIGURE 6:**
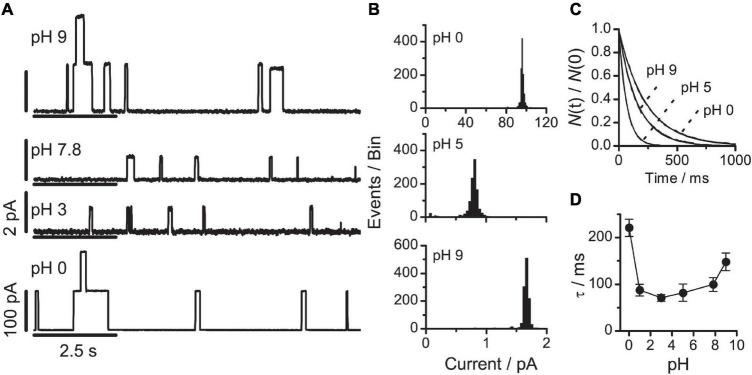
VgA(15) channel function in DOPE:DOPC(4:1)/*n*-decane bilayers at different pHs **(A)** single-channel current traces at pH: 9, 7.8, 3, or 0. The current calibration bars are 2 pA at pH 9, 7.8 and 3, and 100 pA at pH 0. **(B)** Current transition amplitude histograms at pH 9; 5 or 0 (results from single experiments). **(C)** The corresponding normalized single-channel duration histograms: at pH 9 (τ = 162 ms); at pH 5 (τ = 65 ms); and at pH 0 (τ = 235 ms). Results from single experiments fitted by single exponential distributions. **(D)** Relation between pH and channel lifetime (mean ± SD, *n* ≥ 3). 0.1 M NaCl buffered with 10 mM Gly-Gly or Na_2_HPO_4_, 200 mV, 100 Hz.

It is difficult to do these titration experiments in DOPE/*n*-decane bilayers, because the transition temperature for the lamellar→H_*II*_ phase transition of DOPE is ∼5^°^C in the presence of hydrocarbon (Kirk and Gruner, 1985). The experiments therefore were done using bilayers formed from DOPE:DOPC (4:1), which was feasible (cf. section “Changing the Phospholipid Head Group Size”) because we avoided pH 6–7, where the bilayers would be unstable. Figure 6 shows current traces and the corresponding current transition amplitude and lifetime distributions. At each pH, only a single channel population was observed (Figures 6A–C).

As expected from the biphasic variation in the head group repulsion, increasing pH from 0 to 9 had a biphasic effect on channel lifetimes. When pH was increased from 5 to 9, τ increased twofold; when pH was decreased from 5 to 0, τ increased threefold (Figure 6D). The single-channel conductance also increased at both alkaline and acid pH; but the conductance increase has different origins in the two pH ranges. At alkaline pH values, where DOPE is negatively charged, the negative surface charge density will increase the interfacial [Na^+^], which will increase the conductance. At acid pH values, both DOPE and DOPC are positively charged, which reduces the interfacial [Na^+^]. Compared to the DOPS experiments, however, the reduction in the interfacial [Na^+^] is more than compensated for by the increased [H^+^], and the increased contribution of H^+^ eventually cause the conductance to increase as seen in the results at pH 0.

### The Changes in Channel Appearance Rate

The above results show that the qualitative effects of changes in lipid head group interactions (be they steric or electrostatic), on gA channel lifetime, can be predicted from the curvature-dependent changes in *F*_dis_ (Eq. 6). A change in $\Delta \,G_{\text{def}}^{\text{D}\to \text{M}}$, and *F*_*dis*_, would be expected to also affect the channel appearance rate. Following Scheme 1, the channel dimerization constant (*K*_*D*_) is given by:


(14) $$K_{\text{D}}=\frac{k_{1}}{k_{-1}}=\exp\left\{\frac{-\Delta \,G^{\text{M}\to \text{D}}}{k_{\text{B}}\,T}\right\}=\exp\left\{\frac{-\left(\Delta \,G_{\text{protein}}^{\text{M}\to \text{D}}+\Delta \,G_{\text{bilayer}}^{\text{M}\to \text{D}}\right)}{k_{\text{B}}\,T}\right\}$$

A change in $\Delta \,G_{\text{def}}^{\text{M}\to \text{D}}$ (and *F*_dis_) will have opposite effects on *k*_1_ and *k*_–1_ and should therefore have qualitatively similar effects on the channel appearance rate (*f* = *k*_1_⋅[M]^2^) and channel lifetime (τ = 1/*k*_–1_). To investigate whether this is the case, we compared the amount of gA added to the aqueous solution to maintain *f* similar in all experiments (the appearance rate varies from experiment to experiment; we aim for *f* ≈ 1/s). The different concentrations that were used in the different experimental conditions are summarized in Table 2.

**TABLE 2:** Changes in gA channel function and the amount of gA added to achieve *f* ≈ 1/s.

Lipid, electrolyte	g ± SD (pS)	τ ± SD (ms)	[gA] (pM)
**DOPS membranes**			
0.1 M Na^+^, pH 8 (Na_2_HPO_4_)	21.4 ± 0.3	1,380 ± 80	0.425
0.1 M Na^+^, pH 7 (Na_2_HPO_4_)	22.6 ± 0.6	1,320 ± 70	0.25
0.1 M Na^+^, pH 7 (HEPES)	23.8 ± 0.4	1,340 ± 50	0.125
0.1 M Na^+^, pH 6 (Na_2_HPO_4_)	23.1 ± 0.6	920 ± 40	0.275
0.1 M Na^+^, pH 5 (Gly-Gly)	21.1 ± 0.3	800 ± 40	0.75
0.1 M Na^+^, pH 4 (Gly-Gly)	20.5 ± 0.2	270 ± 30	1.75
0.1 M Na^+^, pH 3 (Gly-Gly)	20.6 ± 0.3	170 ± 10	5
DOPS:DOPE (1:2), 0.1 M Na^+^, pH 7 (HEPES)	15.2 ± 0.3	140 ± 20	3.5
DOPS:DOPC (1:2), 0.1 M Na^+^, pH 7 (HEPES)	15.0 ± 0.4	470 ± 50	0.75
**DOPE:DOPC (4:1) membranes**			
0.1 M Na^+^, pH 9 (Na_2_HPO_4_)	8.4 ± 0.4	150 ± 20	3.75
0.1 M Na^+^, pH 7.8 (Na_2_HPO_4_)	6.0 ± 0.3	100 ± 10	2.25
0.1 M Na^+^, pH 5 (Gly-Gly)	4.0 ± 0.3	80 ± 10	5
0.1 M Na^+^, pH 3 (Gly-Gly)	5.8 ± 0.4	70 ± 5	3.75
0.1 M Na^+^, pH 1	90.2 ± 1.8	90 ± 10	7.5
0.1 M Na^+^, pH 0	480 ± 11	220 ± 15	2.25
DOPC, 1.0 M Na^+^	14.3 ± 0.3	550 ± 90	0.75
DOPE:DOPC (1:3), 1.0 M Na^+^	14.4 ± 0.5	350 ± 50	2.5
DOPE:DOPC (1:1), 1.0 M Na^+^	14.2 ± 0.4	240 ± 20	2.5
DOPE:DOPC (3:1), 1.0 M Na^+^	13.7 ± 0.3	260 ± 50	2.25

Under experimental conditions that decreased τ, it was necessary to add an increased amount of gA in order to maintain comparable appearance rates. For example, when the DOPE:DOPC ratio was increased from 0 to 3:1, which caused a twofold decrease in τ, it was necessary to add threefold more gA in order to maintain *f* ≈ 1/s. This means that *k*_1_ had decreased by about 10-fold (because *f* = *k*_1_⋅[M]^2^). The larger effect on *k*_1_ is expected because channel disappearances involve a monomer separation of only ∼0.16 nm, which is much less than the bilayer deformation associated with channel formation (∼1.8 nm). Going from DOPC to DOPE:DOPC 3:1 bilayers decreased *K*_*D*_ (= *k*_1_⋅τ) some 20-fold, meaning that the free energy change associated with gA channel formation, Δ*G*^M→D^, increased about 8 kJ/mole.

Similar results were obtained in experiments where the pH of the electrolyte solution bathing a DOPS bilayer was varied. The channel lifetime decreased sevenfold when pH was lowered from 7 to 3, and 10- to 20-fold more gA had to be added to maintain a comparable *f* (Table 2). Using the same reasoning as above, this means that *k*_1_ decreased 100–400-fold—and that *K*_*D*_ decreased some 1,000-fold, corresponding to a ∼18 kJ/mole increase in Δ*G*^M→*D*^.

### The Experimental Maneuvers Have Minimal Effect on Bilayer Thickness

*F*_*dis*_ varies as a function of changes in *c*_0_, the elastic coefficients *H*_*B*_ and *H*_*X*_ and the channel-bilayer hydrophobic mismatch, *d*_0_ – *l* (cf. Eq. 6). To probe whether the changes in lifetime are due to altered bilayer thickness, we measured the bilayer capacitance. The capacitance of DOPE:DOPC (3:1)/*n*-decane bilayers was 3% less than that of DOPC/*n*-decane bilayers; the capacitance of DOPS/*n*-decane bilayers at pH 4.0 and 7.0 differed by less than 1%. We conclude that the thickness of these *n*-decane-containing bilayers varies little as we change the head group interactions, consistent with studies on the effect of amphiphiles on the thickness of *n*-decane-containing bilayers (Finol-Urdaneta et al., 2010).

### Changes in Gramicidin Function Are Not Due to Changes in Bilayer Elasticity

In the preceding experiments the consequences of altered lipid head group interactions on gA channel function conform to predictions based solely on the changes in *c*_0_. Because the bilayer elastic moduli are determined by the lateral interactions among the bilayer lipids (Helfrich, 1981) and changes in surface charge in their own right are expected to alter the moduli (Winterhalter and Helfrich, 1988), could the changes in gA channel function be due to changes in bilayer properties other than *c*_0_? We have previously shown that a number of amphiphiles modulate gA channel function in a manner that, in addition to possible effects on *c*_0_, involves changes in the bilayer elastic moduli as reflected in the elastic coefficient *H*_*B*_ (cf. Eq. 6)—and that the functional consequences of the changes in bilayer elasticity dominate the changes in *c*_0_ (Lundbæk et al., 2005; Bruno et al., 2007, 2013). (Both *H*_B_ and *H*_X_ are functions of the bilayer elastic moduli, but changes in *H*_B_ are more readily detected). Do the effects of altered interactions among the lipid head groups observed in the present study involve changes in the elastic coefficients *H*_B_ and *H*_X_? To investigate this question, we examined the effects of altered head group interactions on lifetimes of gA channels of different hydrophobic lengths.

The basis for the experiments is that the disjoining force acting on a gA channel (*F*_*dis*_, Eq. 6) can be expressed as the sum of contributions that vary as a function of the hydrophobic mismatch, 2⋅*H*_*B*_⋅(*d*_0_–*l*), and of the lipid intrinsic curvature, *H*_*X*_⋅*c*_0_. It is possible to vary hydrophobic mismatch, *d*_0_–*l*, by doing experiments with gA channels of different lengths. It thus becomes possible to determine whether a change in membrane composition (or head group interactions) alters the elastic coefficient *H*_B_ (and therefore the bilayer elastic moduli). Combining Eqs 12 and 13, τ can be expressed as:


(15) $$\ln\left\{\tau \right\}=\ln\left\{\tau_{0}\right\}+\frac{\Delta \,G_{\text{protein}}^{‡,D\to \text{M}}+\Delta \,G_{\text{bilayer}}^{‡,D\to \text{M}}}{k_{\text{B}}\,T}=\ln\left\{\tau_{0}\right\}+\frac{\Delta \,G_{\text{protein}}^{‡,D\to \text{M}}}{k_{\text{B}}\,T}-\delta \cdot \frac{2\cdot H_{\text{B}}\cdot \left(d_{0}-l\right)+H_{\text{X}}\cdot c_{0}}{k_{\text{B}}\,T}.$$

Assuming τ_0_ and $\Delta \,G_{\text{protein}}^{‡,D\to \text{M}}$ do not vary as we alter the head group interactions, the changes in τ caused by changes in pH (in DOPS membranes) or increases in the mole fraction of DOPE (in DOPE:DOPC membranes) will be given by Rusinova et al. (2011):


(16) $$\frac{\tau }{\tau^{\text{ref}}}=\exp\left\{-2\cdot \delta \cdot \frac{H_{\text{B}}\cdot \left(d_{0}-l\right)-H_{\text{B}}^{\text{ref}}\cdot \left(d_{0}^{\text{ref}}-l\right)}{k_{\text{B}}\,T}\right\}\cdot \exp\left\{-\delta \cdot \frac{H_{\text{X}}\cdot c_{0}-H_{\text{X}}^{\text{ref}}\cdot c_{0}^{\text{ref}}}{k_{\text{B}}\,T}\right\},$$

where the superscript “ref” denotes the reference situation. The ratio of the changes in τ observed with channels that differ in length, e.g., channels formed by 15- and 13-amino acid residue gA analogs, therefore will be given by:


(17) $$\frac{\tau_{2}\cdot \tau_{1}^{\text{ref}}}{\tau_{2}^{\text{ref}}\cdot \tau_{1}}=\exp\left\{-2\cdot \delta \cdot \frac{\left(H_{\text{B}}-H_{\text{B}}^{\text{ref}}\right)\cdot \left(l_{2}-l_{1}\right)}{k_{\text{B}}\,T}\right\}$$

where the subscripts “1” and “2” denotes the channels of different length. That is, when a change in τ is caused by an experimental maneuver that produces a change in *H*_B_, the relative change in τ will differ for channels of different lengths. (The linear relation between lnt and *d*_0_ applies to both nominally hydrocarbon-free and hydrocarbon-containing bilayers; Lundbæk and Andersen, 1999, and Eq. 16 should apply quite generally).

**FIGURE 7:**
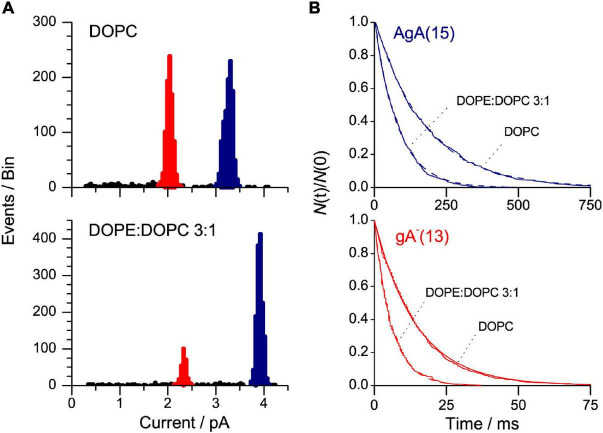
AgA(15) and gA^–^(13) channel function in DOPE:DOPC/*n*-decane bilayers. **(A)** Current transition amplitude histograms. The lower amplitude peaks, highlighted in red, denote gA^–^(13) channels; the higher amplitude peaks, highlighted in blue, the AgA(15) channels. **(B)** The corresponding normalized single-channel duration histograms (the colors have same significance as in **(A)**. The results from single experiments fitted by single exponential distributions. 1 M NaCl, pH 7, 200 mV, 500 Hz.

Figure 7 shows current transition amplitude and lifetime distribution histograms from experiments with channels formed by two different gA analogs, the 15-amino acid gA analog AgA(15) and the 13-amino acid analog gA^–^(13), to DOPC or DOPE:DOPC (3:1) bilayers. [AgA(15) and gA^–^(13) have opposite chirality in order to preclude the formation of heterodimers (Hwang et al., 2003), which considerably simplifies the analysis].

**FIGURE 8:**
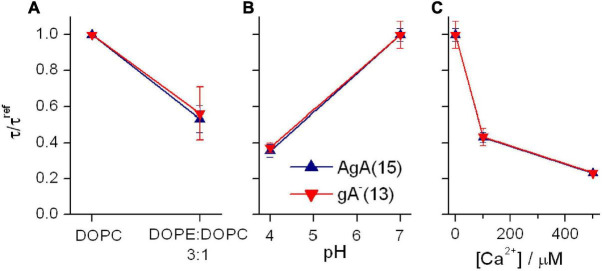
Effects of changes in bilayer composition and head group interactions on AgA(15) and gA^–^(13) channel lifetimes; results for AgA(15) in blue, results for gA^–^(13) in red. **(A)** Effects of varying the DOPE:DOPC molar ratio. The superscript “ref” denotes the value in DOPC bilayers. Experimental conditions as in Figure 7. **(B)** Effects of pH on the channel lifetimes in DOPS bilayers. The superscript “ref” denotes the value at pH 7. 0.1 M NaCl. **(C)** Effects of [Ca^2+^] on the channel lifetimes in DOPS bilayers. The superscript “ref” denotes the value in the nominal absence of Ca^2+^. 0.1 M NaCl, pH 7. In all panels: mean ± SD, *n* ≥ 3.

The presence of DOPE decreases τ for both channel types, irrespective of the channel helix sense—and the relative change in τ is the same for the shorter gA^–^(13) and the longer AgA(15) channels (Figure 8A). These results show that the DOPC→DOPE substitution has little effect on H_*B*_—meaning that the changes in τ do not result from altered bilayer elastic moduli.

We similarly examined the consequences of changing the electrostatic interactions among lipid head groups in DOPS bilayers (Figure 8B). When the aqueous pH on both sides of the membrane was decreased from 7 to 3, the lifetime of channels formed by either AgA(15) or gA^–^(13) decreased—by the same factor (Figure 8B). When the aqueous [Ca^2+^] on both sides of the bilayer was increased, from 0 to 500 μM, the lifetime of both channel types again decreased by similar factors (Figure 8C).

### Lipid Bilayer Material Properties vs. Phospholipid-Channel Interactions

Experimental maneuvers that alter head group interactions and lipid intrinsic curvature may alter other bilayer properties, such as head group hydration (Elworthy, 1962; Rand and Parsegian, 1989)—and maybe more specific gA-phospholipid interactions that could alter gA channel function (Scarlata and Gruner, 1997; Rostovtseva et al., 1998). To address the possible importance of specific gA-lipid interactions, we note that gA channel function in bilayers formed by the ether phospholipid DoPC does not depend on the chirality of the bilayer-forming lipids or the gA channels (Providence et al., 1995). (Hydrocarbon-free, DOPC and DoPC bilayers have similar thickness and cross-sectional area/molecule; Rostovtseva et al., 1998; Guler et al., 2009). Moreover, the DOPC→DoPC change in bilayer composition causes similar increases in channel lifetime for both VgA(15) channels and the tetra-Trp→Phe-substituted [Phe^9,11,13,15^]gA (or gM Heitz et al., 1982; Providence et al., 1995), irrespective of channel helix sense. These results, together with the results on channels of different chirality and lengths in Figures 7, 8, and the similar effects observed with AgA(15) and VgA(15) channels (cf. Figures 3, 7, 8), largely exclude that the changes in channel function we observe are due to specific channel-lipid interactions.

**FIGURE 9:**
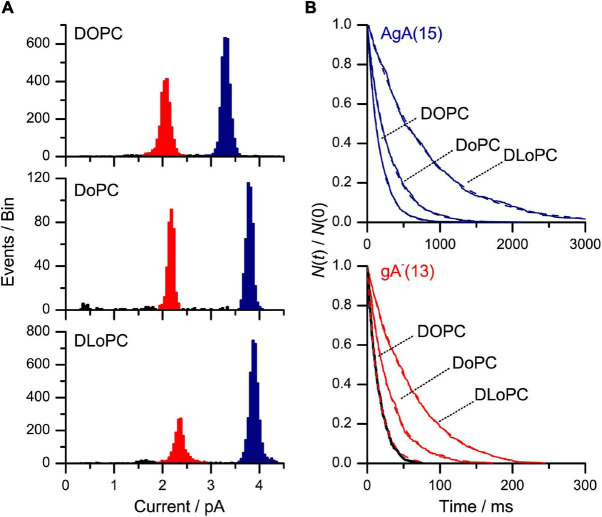
Effects of changes in bilayer composition on AgA(15) and gA^–^(13) channel function. **(A)** Current transition amplitude histograms obtained in (from top to bottom): DOPC/*n*-decane, DoPC/*n*-decane and DLoPC/*n*-decane bilayers. The lower amplitude peaks, highlighted in red, denote gA^–^(13) channels; the higher amplitude peaks, highlighted in blue, the AgA(15) channels. **(B)** The corresponding normalized single-channel duration histograms. Results from single experiments fitted by single exponential distributions (the colors have same significance as in **A**). The lifetime results are summarized in Table 3. 1 M NaCl, pH 7, 200 mV, 500 Hz.

The longer single-channel lifetimes in DoPC/*n*-decane, as compared to DOPC/*n*-decane, bilayers could reflect more specific gA-phospholipid interactions (Rostovtseva et al., 1998) or, maybe, changes in bilayer elasticity because the bending modulus of bilayers formed by ether phospholipids is less than that of bilayers formed by the corresponding ester phospholipids (Guler et al., 2009). We probed this question using the same strategy as in Figures 7, 8. Both the shorter gA^–^(13) and the longer AgA(15) channels had longer lifetimes in DoPC bilayers as compared to DOPC bilayers (Figure 9), and the relative lifetime increase was larger for the shorter gA^–^(13) channels (Figure 9 and Table 3).

**TABLE 3:** gA channel lifetimes in bilayers formed by DOPC, DoPC, and DLoPC.

	τ_13_ (Mean ± SD)	τ_15_ (Mean ± SD)	τ_13_/τ_15_ (Mean ± SE)	*n*
DOPC	13.8 ± 2.6 ms	170 ± 19 ms	0.081 ± 0.003	20
DoPC	29 ± 3 ms	296 ± 29 ms	0.099 ± 0.009	3
DLoPC	61 ± 8* ms	823 ± 84* ms	0.074 ± 0.002*	2

τ_13_ and τ_15_ denotes the lifetimes of gA^–^(13) and AgA(15) channels. *Mean ± range.

We conclude, cf. Eq. 16, that the increased lifetimes in the DoPC bilayers reflect an increased bilayer elasticity (decrease in *H*_B_), rather than specific gA-phospholipid interactions. Based on the results in Table 3 and using Eq. 16, we estimate the decrease in *H*_*B*_ to be − 5 kJ/(mol⋅nm^2^)—a change of about 10% of the value for *H*_*B*_ in mono-unsaturated phospholipid/*n*-decane bilayers [∼50 kJ/(mol⋅nm^2^) as estimated from results in Hwang et al., 2003, see also Rusinova et al., 2011].

### Acyl Chain-Dependent Changes in *c*_*0*_

We finally note that the predictable effects of changing *c*_0_ are limited to maneuvers that involve changes in head group interactions. Increasing acyl chain unsaturation cause a more negative intrinsic curvature, presumably due to an increased range of thermal motion (Tate et al., 1991; Feller et al., 2002), but the lifetimes of channels formed by VgA(15) and analogs with quadruple Trp-aromatic substitutions (in which the Trp was replaced by either Phe, Tyr or naphthylalanine) are four to fivefold longer in bilayers formed using the di-unsaturated DLoPC as compared to DOPC/*n*-decane bilayers (Girshman et al., 1997, see also Bruno et al., 2013).

Using the same strategy as in Figures 7, 8, the lifetimes of both the shorter gA^–^(13) and the longer AgA(15) channels were increased in DLoPC (relative DOPC) bilayers (Figure 9), though the relative increase in τ for gA^–^(13) channels ( $\tau_{\text{DLoPC}}^{13}/\tau_{\text{DOPC}}^{13}$) was less than the relative increase in τ for AgA(15) channels ( $\tau_{\text{DLoPC}}^{15}/\tau_{\text{DOPC}}^{15}$) (Table 3), contrary to our expectation that $\tau_{\text{DLoPC}}^{13}/\tau_{\text{DOPC}}^{13}$> $\tau_{\text{DLoPC}}^{15}/\tau_{\text{DOPC}}^{15}$. These results could be interpreted as the dual consequences of changes in *c*_0_ and elasticity (e.g., May, 2000): the more negative *c*_0_ that results from the increased acyl chain unsaturation (Tate et al., 1991; Talbot et al., 1997) would be expected to produce the same increase of *F*_*dis*_ (and relative decrease in τ) for gA^–^(13) and AgA(15) channels (Eq. 6); poly-unsaturation decreases the bilayer bending modulus (Rawicz et al., 2000), which would be expected to produce a larger increase in $\tau_{\text{DLoPC}}^{13}/\tau_{\text{DOPC}}^{13}$ than in $\tau_{\text{DLoPC}}^{15}/\tau_{\text{DOPC}}^{15}$.

### Experiments With Hydrocarbon-Free Membranes

**FIGURE 10:**
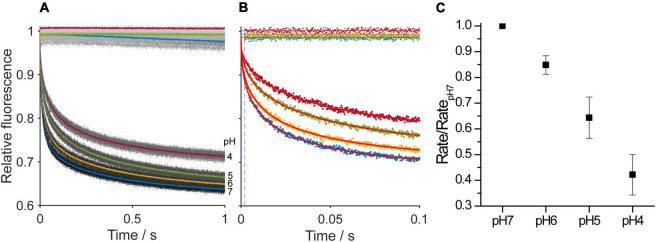
Changes in the time course of fluorescence quench of ANTS-loaded LUVs without gramicidin or that were doped with gramicidin as a function of pH. **(A)** One second fluorescence quench traces, where the dots denote results from individual repeats (*n* > 5) and the heavier traces the average over all repeats in the experiment. **(B)** One hundred millisecond traces, where the dots denote results from a single repeat and curves are stretched exponential fits to the data. In **(A,B)**, the top traces were recorded in LUVs without gramicidin and the bottom traces were recorded with gramicidin-doped LUVs. **(C)** As the pH was decreased the quench rate decreased, which denotes a decrease in the average number of conducting gramicidin channels in the LUV membranes.

We have shown that increasing the intrinsic curvature of hydrocarbon-containing DOPS planar bilayers by protonating the anionic headgroup reduces gA channel activity. To verify that the observed changes are due to the presence of hydrocarbon we also measured the changes in gramicidin channel function in hydrocarbon-free bilayers using a fluorescence-quench assay, where gramicidin monomers incorporated into fluorophore ANTS-loaded DEPS LUVs dimerize to form channels permeable to the ANTS quencher thallium (Tl^+^). Thallium entry thus results in fluorescence quench, and the quench rate will be proportional to the number of conducting gramicidin channel dimers in the LUV membrane. Changes in lipid curvature will alter the bilayer contribution to the free energy of dimerization (the number of gramicidin dimers) and therefore the quench rate. The rates were quantified and analyzed as described in section “Materials and Methods.” As would be expected from the single-channel experiments, decreasing the extravesicular pH reduced the quench rate (Figure 10), which shows that the time-averaged number of gramicidin dimers has been reduced. The reduction in quench rate is similar to the pH-dependent decreases in single-channel lifetimes and appearance rates we observe in hydrocarbon-containing planar lipid bilayers, meaning that negative changes in curvature reduces gramicidin channel activity (increases $\Delta \,G_{\text{bilayer}}^{M\to D}$) in both hydrocarbon-containing and hydrocarbon-free bilayers.

## Discussion

Gramicidin channel function is modulated by experimental maneuvers that alter steric or electrostatic interactions among the head groups of the bilayer-forming lipids. The relation between changes in head group interactions and in channel function is, perhaps surprisingly, simple; a decrease in the effective head group size—caused by a change in either steric or electrostatic head group interactions—decreases both channel appearance rate and lifetime, and *vice versa*. These functional changes conform to predictions based on the changes in lipid intrinsic curvature. Moreover, using the ability to manipulate the gA channel-bilayer hydrophobic mismatch by engineering channels of different length, we find that the relative changes in channel lifetimes do not depend on the channel length. This result shows that the bilayer elastic moduli, as reflected in *H*_*B*_, vary little as we vary the head group interactions, which suggests that the changes in channel function (bilayer deformation energy) are due to changes in *c*_0_.

This relative simplicity breaks down when the curvature is altered by maneuvers that alter the acyl chain interactions, such increasing acyl unsaturation (Figure 9 and Table 3), which may reflect that bilayer elastic moduli and intrinsic curvature are functions of the profile of intermolecular interactions across the lipid bilayer (Helfrich, 1981), meaning that changes in one parameter, such as *c*_0_, may be associated with change in other parameters, such as the elastic moduli (May, 2000), though the contribution may be modest.

We first discuss the possible confounding influence of specific ion-gA channel interactions and conclude that they are not of primary importance in our case. We next consider to what extent the results may be influenced by lipid redistribution in the vicinity of the gA channels, and the use of bilayers formed using *n*-decane. We then consider the quantitative relationship between the changes in gA channel lifetimes and dimerization constant and the changes in electrostatic energy per phospholipid head group and finally the general relations between gA channel function and lipid head group effective size and the implications for the bilayer regulation of integral membrane protein function.

### Changes in Channel Lifetime Are Not Due to Specific Ion-Channel Interactions

When we change the electrostatic interactions among the head groups, we also change the interfacial ion concentrations. Though gA channel lifetimes vary with permeant ion concentration (Ring and Sandblom, 1988; Rostovtseva et al., 1998), the changes in lifetime we observe here are not due to direct ion-channel interactions (see also Lundbæk et al., 1997; Rostovtseva et al., 1998). First, we observe lifetime changes in experiments with net neutral bilayers, where the conductance varies little, such as the DOPC:DOPE experiments. Second, the effects of monovalent (H^+^ or Na^+^) and divalent (Ca^2+^ or Mg^2+^) cations are qualitatively similar, though only the monovalent ions permeate the channel and, at the concentrations used here, Ca^2+^ has no effect on gA channel function in DOPC bilayers (Lundbæk et al., 1997). Third, when [NaCl] is increased from 0.1 to 1 M (at pH 7), the lifetime of channels in DOPS bilayers is decreased twofold, due to increased screening of the head group charge, which reduces the electrostatic repulsion among the head groups, even though there is little change in the single-channel conductance (Lundbæk et al., 1997). Fourth, when [Na^+^] is decreased at constant ionic strength (using the inert tetraethylammonium, TEA^+^), in which case there will be little change in the electrostatic repulsion among the head groups, there is no significant change in channel lifetimes, even though the single-channel conductance *does* decrease (Lundbæk et al., 1997) due to the screening by TEA^+^. Fifth, the quantitative relation between ion-induced changes in τ and bilayer electrostatic free energy is similar for H^+^, Na^+^ Ca^2+^, and Mg^2+^, as will be shown below.

### Effects of Local Lipid Enrichment?

Bilayers formed from lipid mixtures (including mixtures of protonated and deprotonated lipid species) may exhibit lateral phase separation (Knoll et al., 1986; Weinrich et al., 2017). In phase-separated bilayers, gA channels in the different phases would be expected to show heterogeneous behavior (cf. Knoll et al., 1986; Weinrich et al., 2017). In all experiments in the present study, the current transition amplitude and channel lifetime histograms did not provide evidence for a heterogeneous channel population. Further, the visual appearance and electrical properties of the bilayers were “normal” in all cases where stable bilayers could be formed. We conclude that the liquid-crystalline bilayers in the present study have no lateral domain organization.

Even so, in the immediate vicinity of a gA channel, nano-scale lipid de-mixing (e.g., accumulation of lipids with a larger head group size and therefore a more positive intrinsic curvature) could occur if it decreased the bilayer deformation energy sufficiently to compensate for the entropic cost of de-mixing (e.g., Andersen et al., 1992; Bruno et al., 2007; Beaven et al., 2017a). Such nano-scale lateral enrichment has been proposed in other systems (Sperotto and Mouritsen, 1993; Dumas et al., 1997; Grossfield et al., 2006). We cannot exclude that lateral enrichment occurs in the present experiments. If it does, it would not affect the qualitative relation between changes in *c*_0_ and changes in gA channel appearance rate and lifetime.

### Hydrocarbon-Containing Bilayers

The single-channel experiments were done using bilayers formed using *n*-decane, which are softer than nominally hydrocarbon-free bilayers formed using squalene (Lundbæk and Andersen, 1999). For the purpose of these experiments the hydrocarbon-containing bilayers have distinct advantages because changes in phospholipid head group interactions that alter *c*_0_ would be expected to alter *d*_0_ in hydrocarbon-free bilayers because the average cross-sectional area/molecule must be the same across a planar bilayer (Figure 1; see Table 4). These changes in bilayer thickness are minimized with bilayers formed using *n*-decane (see also Finol-Urdaneta et al., 2010).

**TABLE 4:** Thickness and intrinsic curvature of diacyl-PC and -PE bilayers.

Lipid	*d*_0_/nm	*c*_0_/nm^–1^	References
DLPC	2.09		Nagle and Wiener, 1988
DLPE	2.58		Kucerka et al., 2005
DOPC	2.68	–0.115	Nagle and Tristram-Nagle, 2000
DOPE	3.04	–0.351	Gawrisch et al., 1992

In addition, to ensure that the maneuvers that alter gramicidin channel function in hydrocarbon/*n*-decane bilayers also occur in hydrocarbon-free bilayers, we did stopped-flow fluorescence quench experiments using gramicidin-doped dierucoylphosphatidylserine LUVs, which showed that the fluorescence quench rate, a measure of the number of gramicidin channels in the LUV membrane, decreases as the pH is decreased (Figure 10), in agreement with the single-channel results in DOPS/*n*-decane membranes (Figures 4, 5).

### Quantitative Aspects

Our results show that changes in phospholipid head group interactions alter gA channel function. It is possible to obtain more quantitative support for this conclusion by comparing the ion-induced changes in the activation energy for gA channel dissociation in DOPS bilayers to the expected changes in bilayer electrostatic free energy. The bilayer electrostatic free energy (*E*_el_) was calculated using the Gouy-Chapman-Stern theory, considering both screening and ion binding (but not the complications imposed by an embedded gA channel), as described in Supplementary Material.

**FIGURE 11:**
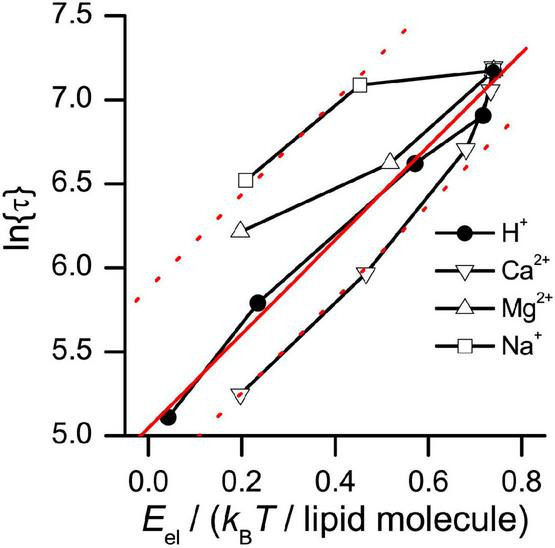
Relation between ln{τ} and the electrostatic free energy (*E*_el_) of DOPS bilayers. *E*_*el*_ was calculated as described in Supplementary Material, using results obtained by either varying the pH (results from Figure 6), or by varying the concentration of Ca^2+^, Na^+^, or Mg^2+^ in aqueous solution, at pH 7 (results from Lundbæk et al., 1997). Solid red line: Linear fit to results obtained using H^+^. Dotted red lines: Linear fit to results obtained using H^+^, shifted vertically to best match results obtained with Ca^2+^ or Na^+^.

Figure 11 shows the relation between *ln*⁡{τ} and *E*_*el*_, where *E*_el_ was varied by changing [H^+^], [Na^+^], [Ca^2+^], or [Mg^2+^] (the latter results are from Lundbæk et al., 1997).

A change in *ln*{τ} provides a measure of the corresponding change in $\Delta \,G_{\text{def}}^{‡,D\to M}$ (cf. Eq. 12) because:


(18) $$\Delta \,\ln\left\{\tau \right\}=\ln\left\{\tau \right\}-\ln\left\{\tau^{\text{ref}}\right\}=\frac{\Delta \,G_{\text{def}}^{‡,D\to \text{M}}-\Delta \,G_{\text{def}}^{‡,D\to M,\mathrm{ref}}}{k_{\text{B}}\,T},$$

where the superscript “ref” denotes the reference situation.

In the experiments where pH was varied, *ln*⁡{τ} is a linear function of *E*_*el*_, with a slope, Δ*ln*⁡τ/Δ*E*_el_≈ 2.8/(*k*_*B*_*T*/lipid molecule) (*r* > 0.99, *p* < 0.0001). In the experiments where [Na^+^] or [Ca^2+^] were varied, *ln*⁡{τ} is a monotonic function of *E*_el_, with a slope similar to that obtained when pH was varied. In the experiments where [Mg^2+^] was varied, the ln{τ} vs. *E*_*el*_ relation is monotonic with a slope that is ∼50% of that obtained when pH, [Na^+^] or [Ca^2+^] was varied. From the slopes of the ln{τ} vs. *E*_*el*_ relation, we find that the changes in $\Delta \,G_{\text{def}}^{‡,D\to M}$ correspond to the change in bilayer electrostatic energy of about three lipid head groups. A gA channel is surrounded by ∼10 lipid molecules in each leaflet [channel radius ≈ 1 nm; phospholipid area/molecule ∼0.7 nm^2^ (Nagle and Tristram-Nagle, 2000) corresponding to a phospholipid radius ≈ 0.5 nm], and the calculated change in *E*_*el*_ for the lipids in the annulus immediately surrounding the channel is about sevenfold (20/3) larger than would be needed to account for the observed changes in $\Delta \,G_{\text{def}}^{‡,D\to \text{M}}$.

A similar conclusion can be reached by examining the relation between the changes in the gA dimerization constant, and Δ*G*^M→D^ (cf. Eq. 14), as deduced from the results in Table 2, and *E*_el_. The changes in Δ*G*^M→D^ are twofold less than the upper limit imposed by the changes in *E*_*el*_. The changes in gA channel function therefore are compatible with the predicted changes in head group interactions.

### Effective Size of Phospholipid Head Groups and Gramicidin Channel Function

Overall, we find that different experimental maneuvers that alter the average effective size of the polar head groups in the host lipid bilayer alter gA channel function as would be predicted from the expected changes in *c*_0_. Though the changes in head group interactions most likely also alter bilayer properties other than *c*_0_, such as the elastic moduli, the results with gA channels of different length (Figures 7, 8) show that any such changes in the bilayer elastic moduli are too small to have measurable effects on channel function.

In contrast, amphiphiles—e.g., capsaicin (Lundbæk et al., 2005), docosahexanoic acid (Bruno et al., 2007), and curcumin (Ingólfsson et al., 2007)—that promote bilayer→non-bilayer (H_II_) phase transitions and cause negative changes in *c*_0_ (Tate et al., 1991; Lundbæk et al., 2005; Barry et al., 2009), turn out to increase, rather than decrease, τ (and correspondingly decrease *F*_*dis*_). These changes in *F*_dis_ most likely arise because reversibly adsorbing amphiphiles for thermodynamic reasons decrease the bilayer elastic moduli (e.g., Evans et al., 1995; Zhelev, 1998; Ly and Longo, 2004; Zhou and Raphael, 2005). Our conclusion that changes in *c*_0_ cause predictable changes in channel function seems to be limited to cases where the changes in *c*_0_ are due to altered head group interactions. Even if the direction of the changes in channel function conform to predictions based on the changes in *c*_0_, as is the case for lysophospholipids (Lundbæk and Andersen, 1994), the magnitude of the changes observed with amphiphiles that can reversibly partition is likely to reflect also changes in bilayer elasticity.

### Implications for Membrane Protein*s*

In this study we focus on gA channels because of their relative simplicity—and the ability to examine the function of channels formed by gA analogs of different lengths, which allows for elucidation of the different contributions to the bilayer deformation energy and for the identification of *c*_0_, *per se*, as an important modulator of gA channels. The results are not specific for gA channels, however, because many different membrane proteins are regulated by maneuvers that change *c*_0_ (Andersen and Koeppe, 2007), even if some of the changes attributed to changes in *c*_0_ may be due to the associated changes in *d*_0_.

Comparing the present results to those obtained on alamethicin channels (Keller et al., 1993; Bezrukov et al., 1998), experimental maneuvers that increase gA channel life times and appearances rates reduce the probability of the higher conductance states in alamethicin channels, whether we change the head group size or the average charge. Given the very different structures of these channels (Woolley and Wallace, 1992), we conclude that they both “sense” similar changes in bilayer properties and that these changes will be pertinent also to bilayer-spanning proteins, though the mechanistic interpretation may be simpler in the case of the gA channels. The same physical principles that pertain to the regulation of gA and alamethicin channel function therefore should apply also to the regulation of integral membrane protein function by the lipid bilayer. A key conclusion in this context is that it will be important distinguish between changes in intrinsic curvature caused by the addition of small molecules, which will alter both curvature and elasticity (e.g., Lundbæk et al., 2005), and by altering phospholipid head group interactions, which alter primarily the curvature.

Though membrane proteins can be regulated by maneuvers that change the lipid bilayer thickness and intrinsic curvature (Andersen and Koeppe, 2007), more specific lipid-protein interactions will be important as well (Lee, 2003, 2004; Marsh, 2008; Landreh et al., 2016; Henault et al., 2019; Cheng et al., 2022), with the prime example being the regulation of many different channels by phosphoinositides (Huang et al., 1998; Hilgemann et al., 2001, 2018; Hansen et al., 2011; Logothetis et al., 2015). Given the chemical heterogeneity of biological membranes (Symons et al., 2021), and the range of lipid specificities of integral membrane proteins (Marsh and Pali, 2004), the challenge becomes to define the relative importance of the more general bilayer and more specific lipid contributions to the free energy difference between different protein conformations, i.e., to the regulation of membrane protein function. A challenge that is amplified when also considering the regulation of membrane protein function by small molecules (Rusinova et al., 2021).

## Data Availability Statement

The data supporting the conclusions of this article will be made available by the authors, without undue reservation.

## Author Contributions

AM and OA designed the study. AM, RR, LP, HI, SC, and JL did the experiments and interpreted the data. AM was the primary author of the manuscript. RR, LP, HI, SC, JL, and OA contributed to the final manuscript. All authors have read and approved the final manuscript.

## Conflict of Interest

The authors declare that the research was conducted in the absence of any commercial or financial relationships that could be construed as a potential conflict of interest.

## Publisher’s Note

All claims expressed in this article are solely those of the authors and do not necessarily represent those of their affiliated organizations, or those of the publisher, the editors and the reviewers. Any product that may be evaluated in this article, or claim that may be made by its manufacturer, is not guaranteed or endorsed by the publisher.
