# Supplementary material for: Regulation of Gramicidin Channel Function Solely by Changes in Lipid Intrinsic Curvature
Source: Front Physiol. 2022 Mar 8;13:836789. doi: 10.3389/fphys.2022.836789 (PMC8957996; doi:10.3389/fphys.2022.836789)
Supplement: Supplementary file 1 [file Data_Sheet_1.PDF]

Regulation of gramicidin channel function by changes in lipid intrinsic curvature

Andreia M. Maer, Radda Rusinova, Lyndon L. Providence, Helgi I. Ingólfsson,

Shemille A. Collingwood, Jens A. Lundbæk and Olaf S. Andersen

Supplementary Material

Calculating the electrostatic energy of a charged phospholipid bilayer

Following [1] we derive a general expression for the free energy of a planar, negatively charged surface that can bind cations, which we then use to consider two cases: first, two monovalent ions, e.g., Na^+ and H^+, compete for binding to charged groups at the bilayer/solution interface; and second, one monovalent and one divalent, e.g., Na^+ and Ca^{2+}, compete for binding to charged groups at the bilayer/solution interface.

1 General Considerations

When $d\Gamma_i$ moles per unit area of a potential-determining (cat)ion of type *i* partition from the bulk electrolyte to the surface, the change in surface free energy per unit area (df) is given by:

$$df = \sum_{i=1}^{n} (\mu_i^S - \mu_i^B) d\Gamma_i \tag{1}$$

where μ_i^S and μ_i^B denote the electrochemical potential of ion *i* at the surface and in the bulk solution. (Ion adsorption will not alter the bulk concentrations measurably, and the bulk electrochemical potential (μ_i^B) is constant.) μ_i^S can be expressed as the sum of a chemical and an electrical contribution:

$$\mu_i^S = \bar{\mu}_i^S + z_i e \cdot \psi^D , \tag{2}$$

where ψ^D is the mean electrostatic potential at the surface (due to the diffuse double layer), z_i the valence of ion *i*, *e* the elementary charge, and $\bar{\mu}_i^S$ the chemical contribution to μ_i^S. Eq. 2 defines $\bar{\mu}_i^S$ and provides a formal separation of the electrostatic and non-electrostatic contributions to μ_i^S.

When ions bind to the surface, the surface charge density (and the surface potential) change and the change f (Eq. 1) is obtained by integrating df with respect to the change in surface charge (σ). Combining Eqs. 1 and 2 :

$$f = \int_{\sigma_i}^{\sigma_f} \psi^D(\sigma) d\sigma + \sum_{i=1}^{n} \int_{initial}^{final} (\bar{\mu}_i^S - \mu_i^B)\, d\Gamma_i , \tag{3}$$

where σ_i and σ_f denote the surface charge densities of the initial state (usually $\sigma_i = 0$) and final (usually the equilibrium) states, and the two integrals in Eq. 3 represent the electrical and the chemical contributions to *f*.

To evaluate the first integral in Eq. 3 we need to know Ψ^D as a function of σ. Using the Gouy-Chapman theory of the diffuse double layer, e.g., [2], the Ψ^D-σ relation becomes:

$$\sigma^2(\psi^D) = 2\cdot\varepsilon_0\varepsilon_r\cdot k_B T\sum_{i=1}^{n} C_i\cdot\left[\exp\{-z_i e\psi^D / k_B T\} - 1\right] \tag{4}$$

where ε_0 and ε_r denote the permittivity of free space and the relative dielectric constant, respectively, k_B Boltzmann's constant, T the temperature in Kelvin, and C_i the bulk concentration of ion type *i*.

To evaluate the second integral we need to know $\bar{\mu}_i^S$ as a function of Γ_i, i.e. the binding isotherm of (cat)ion type *i* to the phospholipid head groups, where we assume the binding isotherm is a stoichiometric Langmuir isotherm and that $\bar{\mu}_i^S$ does not depend explicitly on ψ^D. Following [1], it becomes helpful to introduce the function $\psi^S(\sigma)$

$$\psi^S(\sigma) = -(\bar{\mu}_i^S - \mu_i^B)/z_i e, \tag{5}$$

such that the equilibrium value of σ (σ_0) can be expressed as a function of the equilibrium values of either ψ^D or ψ^S:

$$\sigma(\psi_0^S) = \sigma(\psi_0^D) = \sigma_0 \tag{6}$$

The total amount of bound charge is

$$\sigma = \sum_{i=1}^{n} z_i e\cdot\Gamma_i, \tag{7}$$

and combining Eqs. 3, 5 and 7 we obtain

$$f = \int_{\sigma_i}^{\sigma_f}\left[\psi^D(\sigma) - \psi^S(\sigma)\right]d\sigma, \tag{8}$$

which forms the basis for the remaining calculations.

Chan and Mitchell (2) analyzed the situation where a single ion type can bind to a monolayer. We need to calculate the free energy for the case where two different cations can bind. We treat two cases: first, the case of two monovalent cations; second, the case of one monovalent and one divalent cation.

2 Two Monovalent Cations

Consider two monovalent cations, e.g. Na^+ and H^+, that can bind to a negatively charged bilayer and compete for the same set of binding sites, and a single anion, e.g. Cl^-. The binding reactions are given by:

$$P^- + Na^+ \leftrightarrow PNa;\ \text{association constant } K_{Na},$$

$$P^- + H^+ \leftrightarrow PH;\ \text{association constant } K_{H}.$$

$[P^-]$, [PNa] and [PH] denote the surface concentrations of negatively charged lipid molecules (no bound a cation), and lipid molecules with a bound Na^+ and lipid molecules with a bound H^+:

$$\begin{aligned}[\text{PNa}] &= K_{\text{Na}} \cdot [\text{P}^-] \cdot [\text{Na}^+]_0 \\ [\text{PH}] &= K_{\text{H}} \cdot [\text{P}^-] \cdot [\text{H}^+]_0\end{aligned} \tag{9}$$

$$\begin{aligned}[\text{P}]_{\text{tot}} &= [\text{P}^-] + [\text{PNa}] + [\text{PH}] \\ \sigma &= -e \cdot [\text{P}^-]\end{aligned} \tag{10}$$

where P_{tot} is the total surface concentration of all types of lipid molecules. $[Na^+]_0$ and $[H^+]_0$ the aqueous cation concentrations at the surface, which are related to the respective bulk concentrations:

$$\begin{aligned}[\text{Na}^+]_0 &= [\text{Na}^+] \cdot \exp\left(-e\psi^{\text{S}} / k_{\text{B}}T\right) \\ [\text{H}^+]_0 &= [\text{H}^+] \cdot \exp\left(-e\psi^{\text{S}} / k_{\text{B}}T\right)\end{aligned} \tag{11}$$

The maximum surface charge density, σ_{max}, is

$$\begin{aligned}\sigma_{\max} &= -e\cdot[\mathrm{P}]_{\mathrm{tot}} \\ &= -e\cdot([\mathrm{P}^-]+[\mathrm{PNa}]+[\mathrm{PH}]) = -e\cdot[\mathrm{P}^-]\cdot(1+K_{\mathrm{Na}}\cdot[\mathrm{Na}^+]_0+K_{\mathrm{H}}\cdot[\mathrm{H}^+]_0)\end{aligned} \quad (12)$$

or

$$\sigma\left(\psi^{\mathrm{S}}\right) = -e\cdot[\mathrm{P}^-] = \frac{\sigma_{\max}}{1+K_{\mathrm{Na}}\cdot[\mathrm{Na}^+]_0+K_{\mathrm{H}}\cdot[\mathrm{H}^+]_0}. \quad (13a)$$

(If the two monovalent cations bind competitively and independently to two different sites, e.g. phosphate and carboxyl groups, Eq. 13 would become:

$$\sigma\left(\psi^{\mathrm{S}}\right) = \sigma_{\max}\cdot\left\{\frac{1}{1+K_{\mathrm{CH}}\cdot[\mathrm{H}^+]_0+K_{\mathrm{CNa}}\cdot[\mathrm{Na}^+]_0}+\frac{1}{1+K_{\mathrm{PH}}\cdot[\mathrm{H}^+]_0+K_{\mathrm{PNa}}\cdot[\mathrm{Na}^+]_0}-1\right\} \quad (13b)$$

where K_{CH}, K_{PH}, K_{CNa} and K_{PNa} denote the association constants for H^+ and Na^+ binding to the carboxyl and phosphate groups, respectively.)

Denoting $[\mathrm{P}]_{\mathrm{tot}}$, [PH] and [PNa] by Γ_{S}, Γ_{H} and Γ_{Na}, respectively, then:

$$\begin{aligned}\sigma_{\max} &= -e\cdot\Gamma_{\mathrm{S}} \\ \sigma(\psi^{\mathrm{S}}) &= -e\cdot(\Gamma_{\mathrm{S}}-\Gamma_{\mathrm{H}}-\Gamma_{\mathrm{Na}}).\end{aligned} \quad (14)$$

Further, introducing the ratio

$$C = \frac{\Gamma_{\mathrm{Na}}}{\Gamma_{\mathrm{H}}} = \frac{[\mathrm{Na}^+]\cdot K_{\mathrm{Na}}}{[\mathrm{H}^+]\cdot K_{\mathrm{H}}} \quad (15)$$

we can express $d\sigma$, $d\Gamma_{\mathrm{H}}$ and $d\Gamma_{\mathrm{Na}}$ as:

$$\begin{aligned} d\sigma &= e\left(1+C\right)d\Gamma_{\mathrm{H}} \\ d\Gamma_{\mathrm{H}} &= \frac{d\sigma}{e\cdot\left(1+C\right)} \\ d\Gamma_{\mathrm{Na}} &= \frac{d\sigma\cdot C}{e\cdot\left(1+C\right)}\end{aligned} \quad (16)$$

Using the relation $\mu_{\mathrm{H}}^{\mathrm{B}} - \bar{\mu}_{\mathrm{H}}^{\mathrm{S}} = \mu_{\mathrm{Na}}^{\mathrm{B}} - \bar{\mu}_{\mathrm{Na}}^{\mathrm{S}} = e\psi^{\mathrm{S}}(\sigma)$, Eq. 8 can be expressed as

$$f = \int_{\sigma_{\mathrm{i}}}^{\sigma_{\mathrm{f}}} \psi^{\mathrm{D}}(\sigma)\, d\sigma - \int_{initial}^{final} (\mu_{\mathrm{H}}^{\mathrm{B}} - \bar{\mu}_{\mathrm{H}}^{\mathrm{S}})\, d\Gamma_{\mathrm{H}} - \int_{initial}^{final} (\mu_{\mathrm{Na}}^{\mathrm{B}} - \bar{\mu}_{\mathrm{Na}}^{\mathrm{S}})\, d\Gamma_{\mathrm{Na}}. \tag{17}$$

To continue, we need the expression for $\psi^{\mathrm{S}}(\sigma)$, which can be obtained from Eqs. 11 and 13a:

$$\sigma(\psi^{\mathrm{S}}) = \sigma_{\max} \Big/ \left[1 + Z \cdot \exp\left(-e\psi^{\mathrm{S}} / k_{\mathrm{B}}T\right)\right] \tag{18}$$

or

$$\psi^{\mathrm{S}}(\sigma) = \frac{-k_{\mathrm{B}}T}{e}\left[\ln\left\{\frac{\sigma_{\max} - \sigma}{\sigma}\right\} - \ln Z\right] \tag{19}$$

where $Z = K_{\mathrm{H}} \cdot [\mathrm{H}^+] + K_{\mathrm{Na}} \cdot [\mathrm{Na}^+] = [\mathrm{H}^+] \cdot K_{\mathrm{H}} \cdot (1+C)$.

Then, by calculating the second term of the integral in Eq. (8), and changing the integrating variable for the first term of the integral from σ toψ^{D}, Eq. (8) can be rewritten as:

$$\begin{aligned} f = &-\int_{\psi^{\mathrm{D}}(\sigma_{\mathrm{i}})}^{\psi^{\mathrm{D}}(\sigma_{\mathrm{f}})} \sigma(\psi^{\mathrm{D}})\, d\psi^{\mathrm{D}} \\ &+\left(\sigma_{\mathrm{f}}\psi^{\mathrm{D}}(\sigma_{\mathrm{f}}) - \sigma_{\mathrm{i}}\psi^{\mathrm{D}}(\sigma_{\mathrm{i}}) - \frac{k_{\mathrm{B}}T}{e} \cdot (\sigma_{\mathrm{f}} - \sigma_{\mathrm{i}}) \cdot \ln\{Z\}\right) \\ &-\frac{k_{\mathrm{B}}T}{e} \cdot \left((\sigma_{\max} - \sigma_{\mathrm{f}}) \cdot \ln\{\sigma_{\max} - \sigma_{\mathrm{f}}\} - (\sigma_{\max} - \sigma_{\mathrm{i}}) \cdot \ln\{\sigma_{\max} - \sigma_{\mathrm{i}}\} + \sigma_{\mathrm{f}} \cdot \ln\{\sigma_{\mathrm{f}}\} - \sigma_{\mathrm{i}} \cdot \ln\{\sigma_{\mathrm{i}}\}\right), \end{aligned} \tag{20}$$

where the integral describes the electrical contribution to the free energy and the remaining terms describe the chemical contribution.

We consider two different approaches to evaluating Eq. 20:

2.1 The Initial State is a Neutral Surface

In this case $\sigma_i = 0$ and $\psi^S = \psi^D = 0$, and the final state, $\sigma_f = \sigma_0$ with the associated values of ψ^S and ψ^D, is reached by dissociation of the potential determining ions. (This approach is useful for comparing the electrical contribution to *f* in different situations.) Eq. 20 then reduces to:

$$f = -\int_0^{\psi_0} \sigma(\psi^D)\, d\psi^D + \sigma_0\psi_0 - \frac{k_B T}{e}\cdot\sigma_0\cdot\left[\ln Z - \ln\left\{\frac{\sigma_{max} - \sigma_0}{\sigma_0}\right\}\right] - \frac{k_B T}{e}\cdot\sigma_{max}\cdot\ln\left\{\frac{\sigma_{max} - \sigma_0}{\sigma_{max}}\right\}$$
$$= -\int_0^{\psi_0} \sigma(\psi^D)\, d\psi^D - \frac{k_B T}{e}\cdot\sigma_{max}\cdot\ln\left\{1 - \frac{\sigma_0}{\sigma_{max}}\right\}, \quad (21)$$

cf., Eq. 3.4 in [1]. Using Eq. 4 and the integration variable $x = \exp\{-e\psi^D/k_B T\}$:

$$f = -\frac{k_B T}{e}\cdot\int_1^{x_0}\sqrt{2\cdot\varepsilon_0\varepsilon_r\cdot RT\cdot\left([\text{Na}^+]+[\text{H}^+]\right)\cdot\left(x + \frac{1}{x} - 2\right)}\frac{1}{x}dx - \frac{k_B T}{e}\cdot\sigma_{max}\cdot\ln\left\{1 - \frac{\sigma_0}{\sigma_{max}}\right\}$$
$$= -\frac{2k_B T}{e}\cdot\sqrt{2\cdot\varepsilon_0\varepsilon_r\cdot RT\cdot\left([\text{Na}^+]+[\text{H}^+]\right)}\cdot\left(x_0 + \frac{1}{\sqrt{x_0}} - 2\right) - \frac{k_B T}{e}\cdot\sigma_{max}\cdot\ln\left\{1 - \frac{\sigma_0}{\sigma_{max}}\right\} \quad (22)$$

where $x_0 = \exp\{-e\psi_0/k_B T\}$, $x_0 > 1$ because $\psi_0 < 0$.

The electrical contribution to the free energy in Eq. 20 is

$$f_{el} = -\frac{k_B T}{e}\cdot\int_1^{x_0}\sqrt{2\cdot\varepsilon_0\varepsilon_r\cdot RT\cdot\left([\text{Na}^+]+[\text{H}^+]\right)\cdot\left(x + \frac{1}{x} - 2\right)}\frac{1}{x}dx + \sigma_0\psi_0$$
$$= -\frac{2\cdot k_B T}{e}\cdot\sqrt{2\cdot\varepsilon_0\varepsilon_r\cdot RT\cdot\left([\text{Na}^+]+[\text{H}^+]\right)}\cdot\left(x_0 + \frac{1}{\sqrt{x_0}} - 2\right) + \sigma_0\psi_0 \quad (23)$$

and the cation binding per surface area is:

$$\Gamma_{Na} = [\text{PNa}] = -K_{Na}\cdot[\text{Na}^+]\cdot\frac{\sigma}{e}\cdot x_0$$
$$\Gamma_H = [\text{PH}] = -K_H\cdot[\text{H}^+]\cdot\frac{\sigma}{e}\cdot x_0. \quad (24)$$

2.2 The Initial State is a Surface With no Bound Ions

In this case $\sigma_i = \sigma_{max}$; the final state is the equilibrium at $\sigma_f = \sigma_0$. (This approach is useful for comparing the chemical contribution to *f*.) Rearranging Eq. 20:

$$\begin{aligned} f &= -\int_{\psi^D(\sigma_{max})}^{\psi_0} \sigma(\psi^D)\, d\psi^D + \sigma_0\psi_0 - \sigma_{max}\psi^D(\sigma_{max}) + \\ &+ \frac{k_B T}{e}\sigma_0 \left[\ln\left\{ \frac{\sigma_{max}-\sigma_0}{\sigma_0} \right\} - \ln\{Z\} \right] + \frac{k_B T}{e}\sigma_{max}\left[\ln Z - \ln\{\sigma_{max}-\sigma_0\} + \ln\{\sigma_{max}\} \right] \\ &= -\int_{\psi^D(\sigma_{max})}^{\psi_0} \sigma(\psi^D)\, d\psi^D + \sigma_{max}\cdot\left[\psi_0 - \psi^D(\sigma_{max}) + \frac{k_B T}{e}\cdot \ln\left\{ \frac{\sigma_{max}}{\sigma_0} \right\} \right] \\ &= \int_{\psi^D(\sigma_{max})}^{\psi_o} \{\sigma_{max} - \sigma(\psi^D)\}\, d\psi^D + \frac{k_B T}{e}\cdot\sigma_{max}\cdot \ln\left\{ \frac{\sigma_{max}}{\sigma_0} \right\} \end{aligned} \tag{25}$$

Again, using Eq. 4 and the integration variable $x = \exp\{-e\psi^D/k_B T\}$:

$$\begin{aligned} f &= -\frac{k_B T}{e}\int_{x(\sigma_{max})}^{x_0} \left[\sigma_{max} + \sqrt{2\varepsilon_0\varepsilon_r RT([\mathrm{Na}^+]+[\mathrm{H}^+])\left(x + \frac{1}{x} - 2 \right)} \right] \frac{1}{x}\, dx + \frac{k_B T}{e}\sigma_{max} \ln\frac{\sigma_{max}}{\sigma_0} \\ &= -\frac{k_B T}{e}\sigma_{max}\left(\ln\frac{x_0}{x(\sigma_{max})} - \ln\frac{\sigma_{max}}{\sigma_0} \right) - \\ &\quad -\frac{2k_B T}{e}\sqrt{2\varepsilon_0\varepsilon_r RT([\mathrm{Na}^+]+[\mathrm{H}^+])}\left(x_0 + \frac{1}{\sqrt{x_0}} - x(\sigma_{max}) - \frac{1}{\sqrt{x(\sigma_{max})}} \right) \end{aligned} \tag{26}$$

3 Monovalent and Divalent ions

We now consider the case of one divalent and one monovalent cation, e.g., Na^+ and Ca^{2+}, which can bind to the negatively charged bilayer. The cations bind to a single type of sites, such that their interaction is competitive. We neglect any contribution from H^+ (which should be justified at pH 7), and Cl^- is the only anion. The binding reactions are described by:

$P^- + Na^+ \leftrightarrow PNa$; association constant K_{Na},

$P^- + Ca^{2+} \leftrightarrow PCa^+$; association constant K_{Ca}.

$$\begin{aligned}&[\mathrm{PNa}]=\Gamma_{\mathrm{Na}}=K_{\mathrm{Na}}\cdot[\mathrm{P}^-]\cdot[\mathrm{Na}^+]_0\\&[\mathrm{PCa}^+]=\Gamma_{\mathrm{Ca}}=K_{\mathrm{Ca}}\cdot[\mathrm{P}^-]\cdot[\mathrm{Ca}^{2+}]_0\\&[\mathrm{P}]_{\mathrm{tot}}=[\mathrm{P}^-]+[\mathrm{PNa}]+[\mathrm{PCa}^+]\\&\sigma=-e\cdot[\mathrm{P}^-]+e\cdot[\mathrm{PCa}^+]=-e\cdot\left(\Gamma_{\mathrm{S}}-\Gamma_{\mathrm{Na}}-2\Gamma_{\mathrm{Ca}}\right)\end{aligned} \tag{27}$$

and

$$\begin{aligned}&\mu^{\mathrm{S}}_{\mathrm{Na}}=\bar{\mu}^{\mathrm{S}}_{\mathrm{Na}}+e\psi^{\mathrm{D}};\quad \mu^{\mathrm{B}}_{\mathrm{Na}}-\bar{\mu}^{\mathrm{S}}_{\mathrm{Na}}=e\psi^{\mathrm{S}}\\&\mu^{\mathrm{S}}_{\mathrm{Ca}}=\bar{\mu}^{\mathrm{S}}_{\mathrm{Ca}}+2e\psi^{\mathrm{D}};\quad \mu^{\mathrm{B}}_{\mathrm{Ca}}-\bar{\mu}^{\mathrm{S}}_{\mathrm{Ca}}=2e\psi^{\mathrm{S}}\end{aligned} \tag{28}$$

Inserting Eqs. 28 in Eq. 1,

$$df=e\psi^{\mathrm{D}}\cdot(d\Gamma_{\mathrm{Na}}+2d\Gamma_{\mathrm{Ca}})-(\mu^{\mathrm{B}}_{\mathrm{Na}}-\bar{\mu}^{\mathrm{S}}_{\mathrm{Na}})d\Gamma_{\mathrm{Na}}-(\mu^{\mathrm{B}}_{\mathrm{Ca}}-\bar{\mu}^{\mathrm{S}}_{\mathrm{Ca}})d\Gamma_{\mathrm{Ca}}=\psi^{\mathrm{D}}(\sigma)d\sigma-\psi^{\mathrm{S}}(\sigma)d\sigma \tag{29}$$

and we recover Eq. 8. Changing the integration variable:

$$f=-\int_{\psi^{\mathrm{D}}_{\mathrm{i}}}^{\psi^{\mathrm{D}}_{\mathrm{f}}}\sigma(\psi^{\mathrm{D}})\,d\psi^{\mathrm{D}}+\sigma_{\mathrm{f}}\psi^{\mathrm{D}}_{\mathrm{f}}-\sigma_{\mathrm{i}}\psi^{\mathrm{D}}_{\mathrm{i}}+\int_{\psi^{\mathrm{S}}_{\mathrm{i}}}^{\psi^{\mathrm{S}}_{\mathrm{f}}}\sigma(\psi^{\mathrm{S}})\,d\psi^{\mathrm{S}}-\sigma_{\mathrm{f}}\cdot\psi^{\mathrm{S}}_{\mathrm{f}}+\sigma_{\mathrm{i}}\cdot\psi^{\mathrm{S}}_{\mathrm{i}} \tag{30}$$

The sum of the first three terms denotes the electrical contribution to free energy; the sum of the last three terms denotes the chemical contribution.

Again it is helpful to consider two different choices of initial conditions. First, however, we need to consider how to find the correct values for σ_0 and ψ_0. Again, we assume the binding of Na^+ and Ca^{2+} is described by the Langmuir isotherm. Similarly to the solution for $\sigma(\psi^S)$ for Na^+ and H^+ (Eqs. 11 and 13), we find (e.g., [3; 4]):

$$\sigma\left(\psi^{\mathrm{S}}\right)=\frac{\sigma_{\max}\cdot\left\{1-K_{\mathrm{Ca}}\cdot[\mathrm{Ca}^{2+}]\cdot\exp\{-2e\psi^{\mathrm{S}}/k_{\mathrm{B}}T\}\right\}}{1+K_{\mathrm{Na}}\cdot[\mathrm{Na}^+]\cdot\exp(-e\psi^{\mathrm{S}}/k_{\mathrm{B}}T)+K_{\mathrm{Ca}}\cdot[\mathrm{Ca}^{2+}]\cdot\exp\{-2e\psi^{\mathrm{S}}/k_{\mathrm{B}}T\}} \tag{31}$$

At equilibrium $\sigma(\psi^S) = \sigma(\psi^D)$ and

$$\sigma\left(\psi^{\mathrm{D}}\right)=\pm\sqrt{2\varepsilon_0\varepsilon_{\mathrm{r}}RT}\cdot\sqrt{[\mathrm{Na}^+]\cdot(x-1)+[\mathrm{Ca}^{2+}]\cdot(x^2-1)+([\mathrm{Na}^+]+2\cdot[\mathrm{Ca}^{2+}])\cdot\left(\frac{1}{x}-1\right)}, \tag{32}$$

where $x=\exp\left(-e\psi^{\mathrm{S}}/k_{\mathrm{B}}T\right)$.

The solution to Eq. 32 involves a seventh-degree polynomial in $x_0 = \exp\left(-e\psi_0^{\mathrm{S}}/k_{\mathrm{B}}T\right)$, which can have more than one real solution. $\sigma(\psi^{\mathrm{D}})$ and ψ^{D} may both be either positive or negative and so may σ_0 and Ψ_0 because Ca^{2+} binds to DOPS with a 1:1 stoichiometry [3; 4]. The surface charge density therefore can change sign at high [Ca^{2+}]. From Eq. 31, $\sigma = 0$ and $\psi = 0$ when [Ca^{2+}] = $\exp\{2e\psi^{\mathrm{S}}/k_{\mathrm{B}}T\}/K_{\mathrm{Ca}}$ (8.33 mM for DOPS bilayers in 0.1 M NaCl). For [Ca^{2+}] lower than this, σ_0 and ψ_0 are negative, for higher [Ca^{2+}] they are positive, and $0 < x_0 < 1$.

Again we consider two different approaches to evaluate Eqs. 31 and 32.

3.1 The Initial State is a Neutral Surface

In this case is $\sigma_{\mathrm{i}} = 0$ and $\psi^{\mathrm{S}} = \psi^{\mathrm{D}} = 0$, the final state, $\sigma_{\mathrm{f}} = \sigma_0$, is reached by dissociation of the potential determining ions. The electrical contribution to the free energy becomes, again changing the integration variable to $x = \exp\{-e\Psi^{\mathrm{D}}/k_{\mathrm{B}}T\}$:

$$f_{\mathrm{el}} = \pm\frac{k_{\mathrm{B}}T}{e}\cdot\sqrt{2\varepsilon_0\varepsilon_{\mathrm{r}}RT}\cdot \int_1^{x_0}\sqrt{[\mathrm{Na}^+]\cdot(x-1)+[\mathrm{Ca}^{2+}]\cdot(x^2-1)+([\mathrm{Na}^+]+2\cdot[\mathrm{Ca}^{2+}])\cdot\left(\frac{1}{x}-1\right)}\cdot\frac{1}{x}dx+\sigma_0\psi_0 \tag{33}$$

with the sign in front of the radical is equal to sign of the surface charge. Cation binding per area unit is described by:

$$[\mathrm{PNa}] = -\frac{1}{1-K_{\mathrm{Ca}}\cdot[\mathrm{Ca}^{2+}]\cdot x_0^2}\cdot\frac{\sigma}{e} \text{ and } [\mathrm{PCa}^+] = -\frac{K_{\mathrm{Ca}}\cdot[\mathrm{Ca}^{2+}]x_0^2}{1-K_{\mathrm{Ca}}\cdot[\mathrm{Ca}^{2+}]x_0^2}\cdot\frac{\sigma}{e}. \tag{34}$$

The total energy density is:

$$f = -\int_0^{\Psi_0}\sigma(\psi^{\mathrm{D}})\,d\psi^{\mathrm{D}} + \int_{\Psi^{\mathrm{S}}(0)}^{\Psi_0}\sigma(\psi^{\mathrm{S}})\,d\psi^{\mathrm{S}} = I_1 + I_2 \tag{35}$$

where $\psi^{\mathrm{S}}(0) = -(k_{\mathrm{B}}T/e)\ln\{1/\sqrt{K_{\mathrm{Ca}}\cdot[\mathrm{Ca}^{2+}]}\}$ (from $\sigma(\psi^{\mathrm{S}}) = 0$).

$$I_1 = \mp\frac{k_\mathrm{B}T}{e}\sqrt{2\varepsilon_0\varepsilon_r RT}\cdot \int_1^{x_0}\sqrt{[\mathrm{Na}^+]\cdot(x-1)+[\mathrm{Ca}^{2+}]\cdot(x^2-1)+([\mathrm{Na}^+]+2\cdot[\mathrm{Ca}^{2+}])\left(\frac{1}{x}-1\right)}\frac{1}{x}dx \tag{36}$$

where the "–" sign is for negative surface charge density.

$$\begin{aligned} I_2 &= \frac{k_\mathrm{B}T}{e}\cdot\sigma_\mathrm{max}\cdot\int_{\frac{1}{\sqrt{K_\mathrm{Ca}\cdot[\mathrm{Ca}^{2+}]}}}^{x_0}\left[\frac{K_\mathrm{Na}\cdot[\mathrm{Na}^+]+2\cdot K_\mathrm{Ca}\cdot[\mathrm{Ca}^{2+}]\cdot x_\mathrm{S}}{1+K_\mathrm{Na}\cdot[\mathrm{Na}^+]\cdot x_\mathrm{S}+2\cdot K_\mathrm{Ca}\cdot[\mathrm{Ca}^{2+}]\cdot x_\mathrm{S}^2}-\frac{1}{x_\mathrm{S}}\right]dx_\mathrm{S} \\ &= \frac{k_\mathrm{B}T}{e}\cdot\sigma_\mathrm{max}\cdot\left[\ln\left\{1+K_\mathrm{Na}\cdot[\mathrm{Na}^+]\cdot x_0+K_\mathrm{Ca}\cdot[\mathrm{Ca}^{2+}]\cdot x_0^2\right\}\right] \\ &-\frac{k_\mathrm{B}T}{e}\cdot\sigma_\mathrm{max}\cdot\left[\ln\left\{\frac{x_0}{\sqrt{K\cdot_\mathrm{Ca}[\mathrm{Ca}^{2+}]}}\right\}-\ln\left\{2+\frac{K_\mathrm{Na}\cdot[\mathrm{Na}^+]}{\sqrt{K_\mathrm{Ca}\cdot[\mathrm{Ca}^{2+}]}}\right\}\right] \end{aligned} \tag{37}$$

where $x_\mathrm{S} = \exp\{-e\psi^\mathrm{S}/k_\mathrm{B}T\}$

3.2 The Initial State is a Surface With no Bound Ions

In this case $\sigma_\mathrm{i} = \sigma_\mathrm{max}$ (with $\sigma(\psi^\mathrm{S}) = \sigma_\mathrm{max}$ such that $\psi^\mathrm{S}(\sigma_\mathrm{max}) = \infty$), and the final state is $\sigma_\mathrm{f} = \sigma_0$ (with $\psi^\mathrm{D}(\sigma_\mathrm{max})$ finite). The equilibrium condition is described by Eq. 6. To circumvent the $\psi^\mathrm{S}(\sigma_\mathrm{max}) = \infty$ problem, it is helpful to calculate f for the initial state being $\sigma_\mathrm{i} = \sigma_\mathrm{max} + \varepsilon$ where σ_max is negative and ε is positive and small. Then one can evaluate f as $f_\varepsilon|_{\varepsilon\to 0}$, where

$$\begin{aligned} f_\varepsilon &= -\int_{\psi^\mathrm{D}(\sigma_\mathrm{max})}^{\Psi_0}\sigma(\psi^\mathrm{D})\,d\psi^\mathrm{D}-\sigma_\mathrm{max}\psi^\mathrm{D}(\sigma_\mathrm{max})+\int_{\psi^\mathrm{S}(\sigma_\mathrm{max}+\varepsilon)}^{\Psi_0}\sigma(\psi^\mathrm{S})\,d\psi^\mathrm{S}+(\sigma_\mathrm{max}+\varepsilon)\psi^\mathrm{S}(\sigma_\mathrm{max}+\varepsilon) \\ &= I_1-\sigma_\mathrm{max}\psi^\mathrm{D}(\sigma_\mathrm{max})+I_2+(\sigma_\mathrm{max}+\varepsilon)\psi^\mathrm{S}(\sigma_\mathrm{max}+\varepsilon) \end{aligned} \tag{38}$$

Introducing the notation $x_\varepsilon = \exp\{-e\psi^\mathrm{S}(\sigma_\mathrm{max}+\varepsilon)/k_\mathrm{B}T\}$ where $\lim x_\varepsilon|_{\varepsilon\to 0} = 0$, and replacing σ with $\sigma_\mathrm{max} + \varepsilon$ in Eq. 30 leads to:

$$x_\varepsilon^2[\mathrm{Ca}^{2+}]K_\mathrm{Ca}(2\sigma_\mathrm{max}+\varepsilon)+x_\varepsilon[\mathrm{Na}^+]K_\mathrm{Na}(\sigma_\mathrm{max}+\varepsilon)+\varepsilon = 0, \tag{39}$$

where we select the solution that satisfies the limit conditions (the one with a minus sign in front of the radical). Expanding the expression for x_ε, the leading terms for $\varepsilon \ll 1$ are:

$$\sqrt{1+A(\varepsilon)\varepsilon} \approx 1+\frac{1}{2}A(0)\varepsilon \text{ and } \frac{A+B(\varepsilon)\varepsilon}{C+D(\varepsilon)\varepsilon} \approx \frac{A+B(0)\varepsilon}{C+D(0)\varepsilon} = \frac{A}{C}\left[1+\left(\frac{B(0)}{A}-\frac{D(0)}{C}\right)\right],$$

and

$$x_\varepsilon \approx -\frac{1}{[\mathrm{Na}^+]K_{\mathrm{Na}}\sigma_{\max}}\varepsilon . \tag{40}$$

The last term in Eq. 38 therefore can be approximated as:

$$\begin{aligned}&(\sigma_{\max}+\varepsilon)\psi^{\mathrm{S}}(\sigma_{\max}+\varepsilon) = -\frac{k_{\mathrm{B}}T}{e}\sigma_{\max}\ln\left\{-\frac{\varepsilon}{[\mathrm{Na}^+]K_{\mathrm{Na}}\sigma_{\max}}\right\}+A(\varepsilon)\\ &\lim A(\varepsilon)\big|_{\varepsilon\to 0} = 0\end{aligned} \tag{41}$$

To evaluate I_2, the third term in f_ε (Eq. 38), it is helpful to use Eq. 32, change the integration variable to $x_{\mathrm{S}} = \exp\{-e\Psi^{\mathrm{S}}/k_{\mathrm{B}}T\}$ and expand the integrand into the two ratios:

$$\begin{aligned}I_2 &= \frac{k_{\mathrm{B}}T}{e}\sigma_{\max}\left[\int_{x_\varepsilon}^{x_0}\frac{[\mathrm{Na}^+]K_{\mathrm{Na}}+2[\mathrm{Ca}^{2+}]K_{\mathrm{Ca}}x_{\mathrm{S}}}{1+[\mathrm{Na}^+]K_{\mathrm{Na}}x_{\mathrm{S}}+2[\mathrm{Ca}^{2+}]K_{\mathrm{Ca}}x_{\mathrm{S}}^2}dx_{\mathrm{S}} - \int_{x_\varepsilon}^{x_0}\frac{1}{x_{\mathrm{S}}}dx_{\mathrm{S}}\right]\\ &= \frac{k_{\mathrm{B}}T}{e}\sigma_{\max}\left[\ln\left\{1+[\mathrm{Na}^+]K_{\mathrm{Na}}x_0+[\mathrm{Ca}^{2+}]K_{\mathrm{Ca}}x_0^2\right\}-\ln x_0\right]\\ &\quad -\frac{k_{\mathrm{B}}T}{e}\sigma_{\max}\left[\ln\left\{1+[\mathrm{Na}^+]K_{\mathrm{Na}}x_\varepsilon+[\mathrm{Ca}^{2+}]K_{\mathrm{Ca}}x_\varepsilon^2\right\}-\ln x_\varepsilon\right]\end{aligned} \tag{42}$$

where $x_0 = \exp\{-e\psi_0/k_{\mathrm{B}}T\}$. Simplifying the expression of f_ε, and let $\varepsilon \to 0$:

$$f = -\sigma_{\max}\psi^{\mathrm{D}}(\sigma_{\max}) - \int_{\psi^{\mathrm{D}}(\sigma_{\max})}^{\psi_0}\sigma(\psi^{\mathrm{D}})\; d\psi^{\mathrm{D}} + \frac{k_{\mathrm{B}}T}{e}\sigma_{\max}\log\left\{\frac{1}{x_0}+[\mathrm{Na}^+]K_{\mathrm{Na}}+[\mathrm{Ca}^{2+}]K_{\mathrm{Ca}}x_0\right\} \tag{43}$$

Using Eq. 4, the first term in Eq. 43 can be evaluated as

$$\sigma_{\max}^2 = 2\varepsilon_0\varepsilon_{\mathrm{r}}RT\left\{[\mathrm{Na}^+](x_{\max}^{\mathrm{D}}-1)+[\mathrm{Ca}^{2+}]((x_{\max}^{\mathrm{D}})^2-1)+([\mathrm{Na}^+]+2[\mathrm{Ca}^{2+}])\left(\frac{1}{x_{\max}^{\mathrm{D}}}-1\right)\right\}, \tag{44}$$

where $x_{\max}^{\mathrm{D}} = \exp\{-e\psi^{\mathrm{D}}(\sigma_{\max})/k_{\mathrm{B}}T\}$, which can be solved for $\Psi^{\mathrm{D}}(\sigma_{\max})$.

It is helpful to separate the second term in Eq. (37) into two integrals: one with integration limits

$\Psi^{D}(\sigma_{max})$ and 0; and one with the limits 0 and Ψ_0. For negative potentials we use the formula for σ with a minus in front of the radical; for positive potentials we use a plus in front of the radical.

To evaluate I_1, the first term in Eq. , we change integration variable, to x, and I_1 becomes:

$$\begin{aligned} I_1 = {} & \frac{k_B T}{e}\sqrt{2\varepsilon_0\varepsilon_r RT}\int_{x_{max}^{D}}^{1}\sqrt{[\mathrm{Na}^+](x-1)+[\mathrm{Ca}^{2+}](x^2-1)+([\mathrm{Na}^+]+2[\mathrm{Ca}^{2+}])\left(\frac{1}{x}-1\right)}\frac{1}{x}dx \\ & -\frac{k_B T}{e}\sqrt{2\varepsilon_0\varepsilon_r RT}\int_{1}^{x_0}\sqrt{[\mathrm{Na}^+](x-1)+[\mathrm{Ca}^{2+}](x^2-1)+([\mathrm{Na}^+]+2[\mathrm{Ca}^{2+}])\left(\frac{1}{x}-1\right)}\frac{1}{x}dx \end{aligned} \tag{45}$$

The sum $I_1 + I_2$ provides the desired expression for f. All the functions are known, and the integrals can be solved (numerically, if not analytically). The integrals were evaluated using Maple (Maplesoft, Waterloo, Canada), to calculate the electrostatic component to free energy per molecule (in k_BT units) for a DOPS bilayer for different cations concentrations, using the following association constants: for Na^+, 0.6 M^{-1} [5]; for Ca^{2+}, 12 M^{-1}; for Mg^{2+}, 8 M^{-1} [4] and for H^+ , ~ 4000 M^{-1} (based on a pK value of the carboxyl group in DOPS of 3.6 in 0.1 M [Na^+] [6].

ACKNOWLEDGMENTS

We Thank Dr, Radu Balan for assistance.

REFERENCES

[1] D.Y. Chan, and D.J. Mitchell, The free energy of an electrical double layer. J. Colloid Interface Sci. 95 (1983) 193-197.

[2] R. Aveyard, and D.A. Haydon, An introduction to the principles of surface chemistry, Cambridge University Press, 1973.

[3] S. McLaughlin, N. Mulrine, T. Gresalfi, G. Vaio, and A. McLaughlin, Adsorption of divalent cations to bilayer membranes containing phosphatidylserine. J. Gen. Physiol. 77 (1981) 445-73.

[4] F. Gambale, A. Menini, and G. Rauch, Effects of calcium on the gramicidin A single channel in phosphatidylserine membranes. Screening and blocking. European biophysics journal : EBJ 14 (1987) 369-74.

[5] M. Eisenberg, T. Gresalfi, T. Riccio, and S. McLaughlin, Adsorption of monovalent cations to bilayer membranes containing negative phospholipids. Biochemistry 18 (1979) 5213-23.

[6] F.C. Tsui, D.M. Ojcius, and W.L. Hubbell, The intrinsic pKa values for phosphatidylserine and phosphatidylethanolamine in phosphatidylcholine host bilayers. Biophys. J. 49 (1986) 459-468.
